# Do genome size differences within *Brachionus asplanchnoidis* (Rotifera, Monogononta) cause reproductive barriers among geographic populations?

**DOI:** 10.1007/s10750-016-2872-x

**Published:** 2016-06-30

**Authors:** Simone Riss, Wolfgang Arthofer, Florian M. Steiner, Birgit C. Schlick-Steiner, Maria Pichler, Peter Stadler, Claus-Peter Stelzer

**Affiliations:** Research Institute for Limnology, University of Innsbruck, Mondseestr. 9, 5310 Mondsee, Austria; Institute of Ecology, University of Innsbruck, Technikerstr. 25, 6020 Innsbruck, Austria; Research Institute for Limnology, University of Innsbruck, Mondseestr. 9, 5310 Mondsee, Austria

**Keywords:** Rotifer, Genome size variation, Hybridization, Speciation, AFLP, Population structure

## Abstract

Genome size in the rotifer *Brachionus asplanchnoidis*, which belongs to the *B. plicatilis* species complex, is greatly enlarged and extremely variable (205–407 Mbp). Such variation raises the question whether large genome size differences among individuals might cause reproductive barriers, which could trigger speciation within this group by restricting gene flow across populations. To test this hypothesis, we used *B. asplanchnoidis* clones from three geographic populations and conducted assays to quantify reproductive isolation among clones differing in genome size, and we examined the population structure of all three populations using amplified fragment length polymorphisms (AFLPs). AFLPs indicated that these populations were genetically separated, but we also found hints of natural gene flow. Clones from different populations with genome size differences of up to 1.7-fold could interbred successfully in the laboratory and give rise to viable, fertile ‘hybrid’ offspring. Genome sizes of these ‘hybrids’ were intermediate between those of their parents, and fitness in terms of male production, population growth, and egg development time was not negatively affected. Thus, we found no evidence for reproductive isolation or nascent speciation within *B. asplanchnoidis*. Instead, our results suggest that gene flow within this species can occur despite a remarkably large range of genome sizes.

## Introduction

**Fig. 1:**
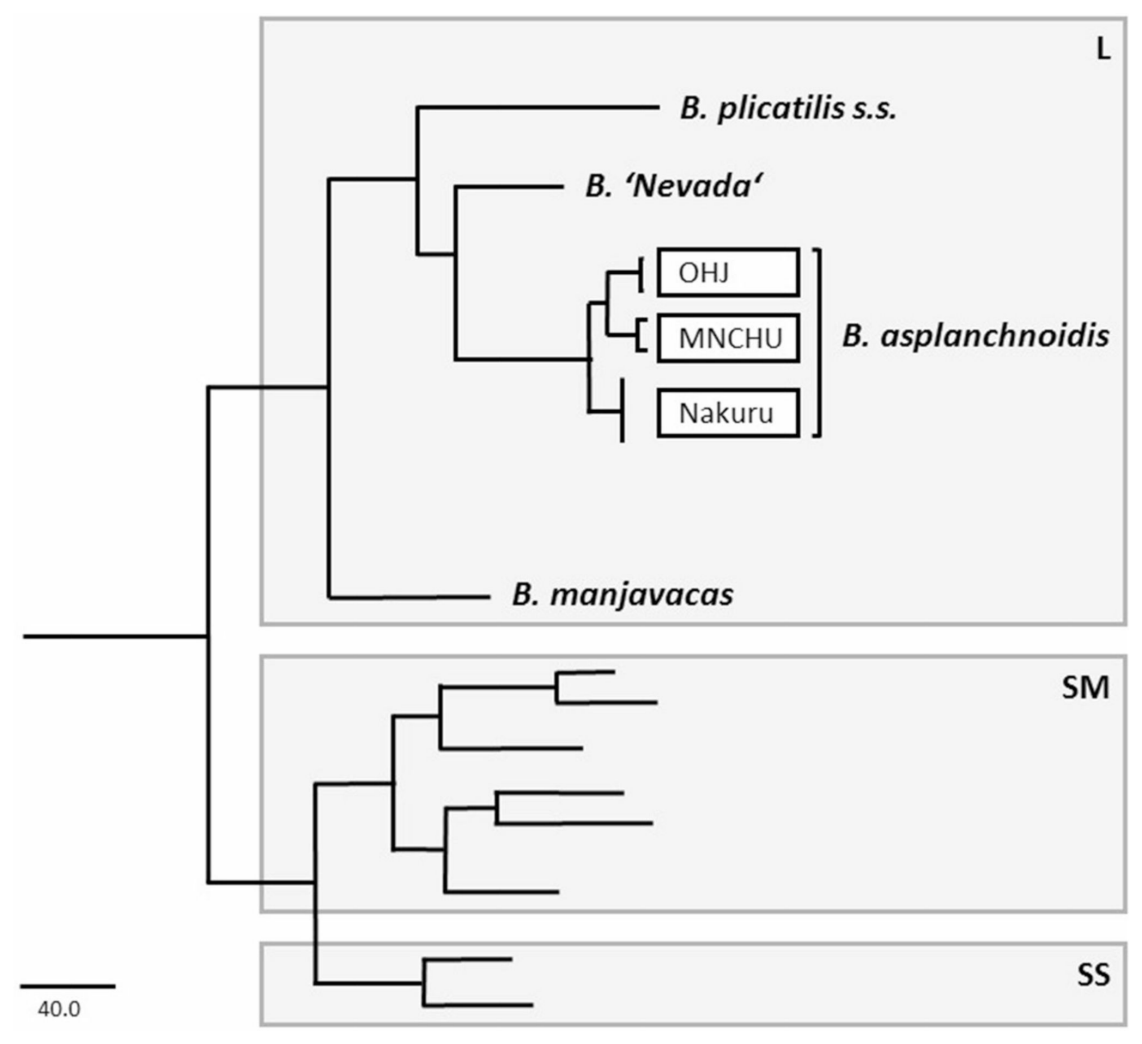
Populations used in this study and their phylogenetic relationship within the *Brachionus plicatilis* species complex. Figure redrawn and simplified from Stelzer et al. (2011). Branch tips represent species, except for *B. asplanchnoidis*. Phylogenetic tree based on the COI and ITS1 Maximum Parsimony tree of Stelzer et al. (2011), which used the same primers as described in our Materials and Methods. Phylogenetic analysis was implemented with PAUP version 4.0 b10 (Swofford, 1998) after multiple alignments with CLUSTAL X. For more details on the procedure, see Stelzer et al. (2011). *Gray boxes* indicate the three major clades (L, SM and SS)

The *Brachionus plicatilis* species complex is an important aquatic invertebrate model system widely used in ecological and evolutionary studies, toxicology, and aquaculture (Lubzens, 1987; Snell & Janssen, 1995). Several features make it particularly suitable for experimental approaches. For example, these rotifers are cyclical parthenogens, i.e., they are capable of clonal reproduction, which allows the same genotypeto be replicated across a large number of treatments. On the other hand, sex can be induced experimentally, which allows to cross different clones with each other (e.g., Fu et al., 1993). Initially considered a single species with geographically variable races, by use of multiple molecular markers *Brachionus plicatilis* Müller, 1786 was shown to be a cryptic species complex of 14–22 species (Gómez et al., 2002; Suatoni et al., 2006; Fontaneto et al., 2009; Gribble & Mark Welch, 2012; Mills et al., 2016). The species complex can be divided into three major clades (see Fig. 1), termed L (large), SM (medium), and SS (small), which show notable differences in body size (Suatoni et al., 2006). These clades are well separated from each other in terms of all species concepts (Suatoni et al., 2006; summarized in Mills et al., 2016) and have probably evolved separately since several millions of years (Gómez et al., 2002).

Within the major clades of the *Brachionus plicatilis* cryptic species complex, species boundaries can differ noticeably depending on the species concept (Suatoni et al., 2006). Cryptic species are commonly detected by the use of phylogenetic methods, e.g., the genealogical species concept (Baum & Shaw, 1995) relies entirely on gene trees to delineate species as genealogically exclusive groups. By contrast, the biological species concept (Mayr, 1963) focuses on successful sexual reproduction as a criterion for species assignment and on reproductive incompatibility to identify diverging groups. However, recognized genealogical species in the *B. plicatilis* complex are often not completely reproductively isolated (Suatoni et al., 2006) and thus, may merge under the biological species concept. The unified species concept aims to accomplish a consensus definition of species by using as the only necessary conceptual property of species the element common to all other species concepts, namely that species are separately evolving metapopulation lineages; in delimiting a particular species, any or multiple of the properties of species considered by other species concepts can then be used as species-delimitation criteria (de Queiroz, 1998, 2007).

Effective reproductive barriers that prevent hybridization are an important condition for the existence of separate species (Arnold, 1997; Rieseberg et al., 2006; Rieseberg & Willis, 2007). Various mechanisms of pre- and postzygotic isolation can inhibit gene flow between groups, thus leading to and maintaining genetic divergence. Several studies have addressed prezygotic and/or postzygotic reproductive isolation among different *B. plicatilis* species (Snell & Hawkinson, 1983; Fu et al.,1993; Hirayama & Hagiwara, 1995; Snell & Stelzer, 2005; Suatoni et al., 2006). They all found strong reproductive isolation between the major clades, varying degrees of reproductive isolation among species within the same clade, and no intraspecific reproductive isolation, except for one case in which behavioral reproductive isolation was observed between two *B. plicatilis* strains (Berrieman et al., 2005). It has been suggested that reproductive isolation between *B. plicatilis* strains from different major clades might be caused by postzygotic reproductive isolation mechanisms such as different karyotypes (Rumengan et al., 1991).

Postzygotic isolation might also be caused by large differences in genome size (Bureš et al., 2009; de Vienne et al., 2009; Hamon et al., 2015). Stelzer (2011) and Stelzer et al. (2011) found high variation in genome size across the *B. plicatilis* complex (haploid ‘1C’ genome sizes: 55–407 million base pairs, Mbp). The majority of the genome size variation within the species complex can be attributed to the three major clades (67%), but considerable variation (32%) also exists at lower taxonomic ranks—within and among genealogical species within the major clades—and, remarkably, even between sister species not yet entirely reproductively isolated. In one genealogical species, *Brachionus asplanchnoidis* Charin, 1947 (in several studies referred to as B. ‘Austria’, see Mills et al., 2016, and Michaloudi et al., 2016, for the rationale of name allocation), genome sizes were extremely variable (205–407 Mbp) and greatly enlarged in relation to the three most closely related sibling species (109–129 Mbp).

In addition to interspecific genome size variation, the same study (Stelzer et al., 2011) detected inter- and intrapopulation variation of genome size in *B. asplanchnoidis*. In an Austrian population, genome sizes range mostly between 205 and 270 Mbp (Stelzer et al., in preparation). Two Mongolian isolates show genome sizes of 340 and 407 Mbp, while *B. asplanchnoidis* populations from the East African Lakes Bogoria and Nakuru have genome sizes of 217-222 Mbp (Stelzer et al., 2011). Such variation in nuclear DNA content raises the question whether speciation processes are currently occurring in *B. asplanchnoidis*. We hypothesized that the large genome size differences between individuals in this species might cause postzygotic reproductive isolation, thus limiting gene flow among populations and providing the ‘seed’ for speciation processes.

In this study, we tested the three main corollaries of this hypothesis: (1) the three populations OHJ (Obere Halbjochlacke, Austria), Nakuru (Lake Nakuru, Kenya), and MNCHU (Chuluutyn Tsagaan Nuur, Mongolia) are reproductively isolated from each other, (2) reproductive isolation is pronounced if the two parents greatly differ in genome size, and (3) past reduction in gene flow among the three populations should have affected their population genetic structure, leaving them as three well-separated groups. The first corollary was addressed using cross-mating experiments, in which we tested whether parents from different populations (differing in genome size) could successfully produce ‘hybrid’ offspring. Postzygotic isolation was examined by measuring the fitness of ‘hybrids’ in terms of embryonic development time, population growth rate, and ability to produce males, to find out whether ‘hybrid’ offspring would exhibit lower fitness due to outbreeding depression (e.g., Tortajada et al., 2010). The second corollary was addressed implicitly in the crossing experiments because we cross-mated parents with small or large differences in genome size. To gain additional insights into the inheritance pattern of the trait ‘genome size,’ we also measured how genome size of ‘hybrid’ offspring differed from their parents. The third corollary was addressed by exploring the population structure of the three populations (OHJ, Nakuru, MNCHU) using amplified fragment length polymorphisms (AFLPs): If these populations have a history of reproductive isolation, they should be genetically well isolated from each other and there should be no signs of interbreeding between populations.

## Materials and methods

### Origin and culture of rotifers

In this study, we used different clones from three *B. asplanchnoidis* populations: OHJ, Nakuru, and MNCHU (see Table 1). A clone is composed of the asexual descendants of one female, which has originally hatched from one resting egg. Members of the same clone are genetically identical, since they were produced asexually, while members of different clones (from the same population) differ from each other in much the same way than the individuals of an obligate sexual species. The OHJ population was sampled from Obere Halbjochlacke, a small alkaline pond near Neusiedlersee (Austria). Our Nakuru population derives from a sediment sample of lake Nakuru (Kenya), while the MNCHU population was originally sampled in Chuluutyn Tsagaan Nuur (Mongolia). We used 86 clones from OHJ population, 15 clones from Nakuru population, and two clones from MNCHU population. The smaller number of clones for Nakuru and MNCHU was due to limited amounts of resting eggs from these populations.

As mentioned above, clonal cultures were established from the hatchlings of individual resting eggs. Rotifers were cultured in F/2 medium (Guillard, 1983) at 16 ppt salinity and with *Tetraselmis suecica* algae as food source at ad libitum concentration (500–1000 cells μl^−1^). Continuous illumination was provided with daylight LED lamps (SunStrip, Econlux) at 30–40 μmol quanta m^−2^ s^−1^ for rotifers and 200 lmol quanta m^−2^ s^−1^ for algae. Clonal stock cultures were kept at 18 °C and were reinoculated once per week by transferring 20 asexual females to fresh culture medium provided in 20 ml petri dishes. Cross-mating experiments, egg development, and population growth assays were conducted at 23–24 °C.

### DNA sequence markers

To obtain rotifer biomass for DNA extraction, clonal cultures with population densities of 10–100 individuals per ml were starved for 16 h, ensuring that rotifers completely emptied their guts of the food algae. Afterward, the washed rotifer biomass was resuspended in 70% ethanol and stored at −20°C. Genomic DNA was isolated using the DNeasy Blood & Tissue kit (Qiagen) according to the manufacturer’s instructions, except that DNA was eluted with 50 μl of 1× TE_0.1_ buffer (20 mM Tris-HCl, 0.1 mM EDTA, pH 8.0). Concentration and quality of DNA was determined using a NanoDrop spectrophotometer (Thermo Scientific), and the DNA samples were additionally run on a 1% agarose gel.

To confirm correct species assignment, we amplified a 661-bp region of the mitochondrial cytochrome c oxidase subunit I (COI) using primers LCO1490 and HCO2198 from Folmer et al. (1994) and a 543-bp segment of the ribosomal internal transcribed spacer 1 (ITS1) using primers III and VIII from Palumbi (1996). PCR reactions were carried out in 20 μl volumes using HotStarTaq Plus Master Mix Kit (Qiagen). Cycling parameters were as follows: one cycle: 95 °C for 5 min; 5 cycles: 94 °C for 40 s, 48 °C (or 45 °C for clone Nakuru8 from population Nakuru) for 40 s, 72 °C for 1 min; 35 cycles: 94 °C for 40 s, 51 °C for 40 s, 72 °C for 1 min; and 72 °C for 10 min. PCR products were purified using the QIAquick PCR Purification Kit (Qiagen) and were sequenced by a commercial sequencing service (Eurofins MWG Operon). COI and ITS1 sequences were aligned using BioEdit 7.2.5 (Hall, 1999). Sequence identities were calculated in BioEdit, and Blastn (http://blast.ncbi.nlm.nih.gov) searches were conducted to confirm the species identity of our clones. Pairwise genetic distances for ITS1 sequences were calculated in MEGA7 (Kumar et al., 2016) using the Maximum Composite Likelihood model (Tamura et al., 2004). All positions containing gaps and missing data were eliminated. There were a total of 539 positions for ITS1 and 661 for COI in the final datasets. New sequences were deposited in the GenBank database (accession numbers for COI: KU299078-KU299174; accession numbers for ITS1: KU299644-KU299740).

### Cross-mating experiments

Cross-mating experiments were used to test for reproductive isolation between clones from the three populations (OHJ, Nakuru, MNCHU). The genome sizes of the parental clones ranged from 211 to 366 Mbp (Table 2). Genome size differences between mating pairs varied between 3 and 155 Mbp, and cross-mating combinations were the following: We crossed females with a rather small genome size from the OHJ population (OHJ22, 211 Mbp) with males with a similar genome size from lake Nakuru (Naku-ru4, 214 Mbp), but also with males with larger genome sizes from Mongolia (MNCHU24, 326 Mbp; MNCHU008, 366 Mbp). Further, we crossed females with a comparably large genome size from the MNCHU population (MNCHU24, 326 Mbp) with males with smaller genome sizes from the OHJ population (OHJ22, 211 Mbp; OHJ7, 264 Mbp). We also tried to cross OHJ7 females with MNCHU24 males and MNCHU008 females with OHJ22 males, but these combinations were not successful (see results).

In the cross-mating experiments, we used freshly hatched virgin females and males, which were harvested as eggs from dense rotifer cultures that had initiated sexual reproduction. Eggs were detached from the females by vigorously vortexing the rotifer culture in 50-ml Falcon tubes for 10 min. Crossings between clones were accomplished by placing 100 female eggs and 50 male eggs together into the same well of a 24-well plate filled with 750 μl of F/2 medium. After 24 h, when all viable eggs had hatched and animals had time to mate, females were transferred to new wells with fresh food suspension. Before these transfers, we checked briefly for mating behavior. The existence of male mating behavior was recorded, if we observed at least two males circling females within a 5-minute interval. To determine the male/female ratios and male population densities in the cross-mating experiments, we also scored the hatching rates of male and female eggs after the 24-h incubation. Upon fertilization, females were cultured for another 8–15 days to classify them as amictic (=asexual) or mictic (=sexual) and to allow fertilized mictic females to produce resting eggs. If females died without producing any eggs, we could not determine their reproductive mode and counted them as undetermined (category “no eggs in” Table 3). Finally, all resting eggs were stored at 7 °C in the dark for at least 2 weeks. To induce hatching, resting eggs were incubated with food suspension at 23 °C and high light intensities (200 μmol quanta m^−2^ s^−1^). Usually after 48 h, the first hatchlings started to emerge, and clonal cultures were initiated. For a maximum of 4 weeks, the remaining resting eggs were checked for hatching. All eggs were transferred to fresh medium at least twice a week.

### Inheritance of genome size

To determine the genome sizes of crossed sexual offspring of different *B. asplanchnoidis* populations and the genome sizes of their parents, we used the flow cytometric method described in Stelzer et al. (2011), which uses propidium iodide (PI) staining of nuclei. Briefly, clonal rotifer populations were grown from low to high population densities in 1-l flasks, which were aerated with sterile air through a glass tube. One day before biomass preparation, animals were collected from each culture using 60-μm nylon sieves, washed in filtered sea water (salinity: 12 ppt), and starved overnight. On the day of biomass preparation, an aliquot of 350 females was taken from each starved culture and was subjected to the flow cytometry protocol of Stelzer et al. (2011). Two modifications of the original protocol were made. First, for each batch of rotifer biomass, we used one head of a female *Drosophila melanogaster* (strain ISO-1, C-value: 0.18 pg according to Gregory, 2015) as an internal standard. This 350:1 ratio yields almost identical peak heights for rotifers and *Drosophila* during flow cytometric analyses. Second, the trypsin digestion step was done at 37 °C in a water bath, rather than at 20 °C as in the original protocol (Stelzer et al., 2011). We found that this modification increased the precision of our flow cytometric measurements, i.e., it resulted in a lower coefficient of variance. The actual measurements were done on an Attune NxT^®^ acoustic focusing cytometer (Thermo Fisher) with an excitation wavelength of 561 nm and a 590–650 nm bandpass filter for detection of PI fluorescence. Flow cytometric data were analyzed using FlowJo software version 10.0.7r2 (FlowJo LLC). At least three replicate measurements were obtained for each rotifer clone, and usually these measurements were done on different days.

### Fitness of ‘hybrid’ offspring

To assess the fitness of ‘hybrid’ offspring in comparison with their parents, we measured three parameters: embryonic development time of amictic eggs, population growth rate, and the ability to produce males. The duration of embryonic development was measured using an automated method described in detail in Stelzer (2016). Briefly, this system relies on time-lapse recording of up to 96 amictic eggs in a simple custom-built inverted microscope with a motorized aperture stage that accepts 96-well plates. Amictic eggs were stripped from females 0–30 min after they had been extruded, and hatching of each individual egg was monitored automatically in 30 min intervals. The temperature during preparation of the eggs and hatching incubations was set to exactly 23.5 °C.

Population growth rates were estimated from exponentially growing rotifer cultures (200 ml culture volume). These cultures had been inoculated at a density of 0.25 females ml^−1^ (=time *T*_0_) and were sampled after three (*T*_1_), four (*T*_2_), and 5 days (*T*_3_) by withdrawing 27-ml culture suspension, followed by fixation with 3 ml Formaline (37%). Population densities at *T*_1_ and *T*_2_ were estimated by counting the complete samples, and at *T*_3_ by counting half of the sample (15 ml) using inverted microscopy. Since our clones differed in their investment into sexual reproduction, we followed the recommendations of Montero-Pau et al. (2014) and calculated the potential intrinsic growth rate, *r*_pot_. Differences in the growth rates between parents and their crossed offspring were statistically analyzed using a general linear model with a hierarchical structure, with “clone” as a random nested variable. All calculations were done in the software package R version 3.2.1 (R Development Core Team, 2015).

The occurrence of sexual reproduction in crossed clones was checked over 3 weeks as part of the serial transfers of our stock cultures. At each weekly transfer, the old culture was closely examined for the presence/absence of males. These assays were intended to detect whether a clone is capable of producing males at all, rather than estimating its sexual propensity.

### Amplified fragment length polymorphisms

Amplified fragment length polymorphisms (Vos et al., 1995) were used to characterize the genetic population structure of *B. asplanchnoidis* populations and to confirm successful cross-mating of clones. In total, we analyzed 127 DNA samples from 103 different *B. asplanchnoidis* clones (86 clones from OHJ, 15 clones from Nakuru, and two from MNCHU) and 11 ‘hybrid’ clones from lab crosses (six intrapopulation [OHJ × OHJ] and five interpopulation ‘hybrids’ [four OHJ × MNCHU, one OHJ × Nakuru]). We also included 13 biological replicates (from separate biomass preparations of the same clones) and a water sample as negative control.

In the restriction-ligation reactions, 100 ng of genomic DNA were digested and ligated to EcoRI-and MseI-adaptors in 11 μl volumes containing 1× CutSmart buffer, 1 mM ATP, 2.5 U MseI, 5 U EcoRI-HF, 1 WeissU T4 DNA ligase (all: New England Biolabs), 20 pmol MseI adaptor, and 2 pmol EcoRI-adaptor. Restriction-ligation reactions were carried out for 3 h at 37 °C followed by 17 °C overnight in a thermal cycler. Afterwards, 5 ll of the DNA were diluted 20-fold with 1× TE_0.1_ buffer. The 20 μl preselective amplification reactions contained 4 μl of the diluted DNA prepared by restriction–ligation, 1× Taq PCR Mastermix (Qiagen), 0.25 lM of each of the two preselective primers (MseI + C and EcoRI + 0), 1.25 mM additional MgCl_2_, and 4.5 μl RNase-free, deionized water (Qiagen). PCR amplification was carried out in a Mastercycler^®^ nexus Gradient (Eppendorf) using the following program: 72 °C for 2 min; 20 cycles: 94 °C for 20 s, 56 °C for 30 s, 72 °C for 2 min; and 60 °C for 30 min. The product was diluted 20-fold with 1× TE_0.1_ buffer. The 10 μl selective PCR reaction contained 2 μl diluted PCR product, 0.25 μM MseI-primer, 0.05 μM fluorescent-labeled EcoRI-primer, 1.25 mM additional MgCl_2_, and 1× HotStar PCR Master Mix (Qiagen). The PCR conditions were as follows: 95 °C for 15 min; 10 cycles: 94 °C for 20 s, 66 °C-1°C/cycle for 30 s, 72 °C for 2 min, 20 cycles: 94 °C for 20 s, 56 °C for 30 s, 72 °C for 2 min; 60 °C for 30 min). We used eight different primer combinations for the selective amplification: M47xE24, M50xE25, M48xE13, M59xE14, M47xE12, M47xE25, M49xE13, and M61xE11 (Nomenclature according to KeyGene, http://www.keygene.com). Core sequences of the primers were EcoRI: 5′-Dye-GACTGCGTACCAATTC-NN-3′and MseI: 5′-GATGAGTCCTGAGTAA-NNN-3′. EcoRI-Primers were labeled with the fluorescent dyes FAM, JOE, Atto550, and Atto565. The most suitable primer combinations were already chosen beforehand using the Selective Amplification Start-Up Kit for Small Plant Genomes (Applied Biosystems). For fragment analysis, 0.5 μl of fluorescent-labeled product was mixed with 10 μl of Hi-Di™ Formamide™ (Applied Biosystems), 10 μl H_2_O, and 0.1 μl LIZ-500 Size Standard (Applied Biosystems), denatured, and analyzed on an ABI 3730 capillary sequencer (Applied Biosystems) by a commercial service (Microsynth).

The fsa files generated during fragment analysis were converted to csv format using PeakScanner v1.0 (Applied Biosystems). optiFLP v1.57 (Arthofer et al., 2011) was used to identify optimum scoring parameters using the following settings: maximum pea-kheight—50, 200, 10 (minimum, maximum, stepwidth); maximum peakwidth—1 (fixed value); minimum peaksize—60, 130, 10; maximum peak-size—250, 400, 10; size tolerance range—0.5; minimum peak distance—off; minimum allele frequency—5, 15, 3; maximum allele frequency—80, 95, 3; Jaccard coefficient; unsupervised mode; lowest group nr.—2; highest group nr.—10; minimum number of profiles per group—2; and paraphyletic groups allowed. optiFLP retains the scoring parameters for the ten runs with highest global R; the run with the absolute maximum of global R was used for further analysis. If two or more runs shared maximum global R considering four decimal places, the run yielding the highest number of loci was selected. The result files of optiFLP were concatenated using tinyCAT v1.2 (Arthofer, 2010).

Dominant marker-based F and θ statistics were computed in Hickory v1.1 (Holsinger et al., 2002). We used a burnin of 50,000 generations, followed by 250,000 sampled generations with a thinning of 5 and the ‘Perform all Analyzes’-option. The deviance information criterion (DIC) was used to identify the best model, but, due to populations containing less than 10 individuals, also the model estimating θ without estimating *f* was considered.

SplitsTree v4.13.1 (Huson & Bryant, 2006) was used to calculate a Neighbor Network with following settings: character transformation—Jaccard; distances transformation—NeighborNet; splits transformation—equal angle. The resulting network visualization was directly exported from SplitsTree as pdf file.

Two Bayesian clustering approaches were used for detecting the true number of clusters (*K*) in our population samples: (i) Structure v2.3.3 (Pritchard et al., 2000) was used with default settings, 20,000 MCMC generations burnin, and 180,000 MCMC generations for data acquisition, with 10 repetitions for each *K* = [1, 7]. Evanno’s delta K algorithm as implemented in Structure Harvester v0.6.94 (Earl & von Holdt, 2011) was used to identify the best *K*. (ii) Clustering of individuals was performed in BAPS v5.3 (Corander et al., 2008) with default settings and 10 repetitions for each *K* = [1, 7]. The same software was used for admixture analysis of individuals based on mixture clustering using the default settings and 50 simulations from posterior allele frequencies. As the number of individuals per population differed largely, and Structure is sensitive to unequal population sizes (Kalinowski, 2011), we generated reduced datasets by randomly deleting 49, 73, 86, and 92 profiles of the OHJ population, with 4 replicates for each reduced set. These sets were analyzed by Structure and BAPS as described above, and optimum values for K were recorded. We also performed the unsupervised, iterative, non-Bayesian clustering method implemented in FLOCK v3.1 (Duchesne & Turgeon, 2009). Settings were 50 runs, 50 iterations per run, and random choice of samples for generating the initial partition. *K* = [2, 7] was searched, and the best value for *K* was determined as suggested in the manual of the software.

## Results

### DNA sequence markers

The sequences of the mitochondrial COI gene and the nuclear ITS1 gene confirmed that all our clones belonged to *B. asplanchnoidis*. The overall sequence diversity of ITS1 waslow: Pairwise genetic distances varied between 0.000000 within populations, 0.003719 between OHJ and MNCHU, 0.003721 between MNCHU and Nakuru, and 0.007460 between OHJ and Nakuru. Within populations, ITS1 sequences were virtually identical (apart from very few cases of clear heterozygous single-nucleotide polymorphisms). Among populations, the ITS1 sequences differed by a maximum of 4 bp resulting in ≥99% shared sequence identity.

COI sequence diversity was also low. Pairwise genetic distances were 0.000000-0.002028 within the Nakuru population, 0.006134 within MNCHU, 0.000000-0.017732 within OHJ, 0.026561-0.029920 between MNCHU and OHJ, 0.037058–0.040539 between MNCHU and Nakuru, and 0.033411–0.042758 between Nakuru and OHJ. Within the OHJ population, we found four different COI haplotypes: three were quite similar to each other with at least 99.6% shared sequence identity (max. 2 base substitutions), but the fourth haplotype showed only 97.4% sequence identity compared with the others. Clones from the Kenyan population Nakuru showed at least 99.6% sequence identity in their COI sequences (max. 2 base substitutions). The previously published COI sequences of the two MNCHU clones are 99.0% identical (6 bp difference). Among populations, the COI sequences were ≥94% identical.

### Cross-mating experiments

In the cross-mating experiments, we tested for reproductive isolation between clones from different populations and with different genome sizes. We examined the presence of male mating behavior, fertilization, the production of resting eggs, and whether resting eggs resulted in viable and fertile offspring (Table 3). Freshly hatched females were exposed to freshly hatched males. The overall hatching rates of female eggs were high and similar in all parental clones (89–97%). In contrast, hatching rates of male eggs were variable: The Mongolian clones exhibited low hatching rates of 3 and 9%, while the male hatching rates of the other *B. asplanchnoidis* clones were similar to their female hatching rates (Table 4). These differences affected the concentrations of males and the male/female ratio in the cross-mating experiments, which may have affected fertilization rates. Further, mixis rates (proportion of sexual females) in the cross-mating experiments were generally low, thus there was only a small number of mictic females that could be fertilized. Accordingly, we did not compare fertilization success between different clone combinations quantitatively, but rather understood the production of one or few viable and fertile F1-offspring as evidence against reproductive barriers.

In all cross-mating combinations, males displayed mating behavior (7 combinations), and in most combinations, females produced resting eggs (5 out of 7 combinations, exceptions: MNCHU008 ♀ × OHJ22 ♂ and OHJ7 ♀ × MNCHU24 #; 2–18 resting eggs per combination), from which viable and mostly fertile offspring hatched (Table 3).

Two mating combinations did not result in the production of resting eggs. In the combination MNCHU008 ♀ × OHJ22 ♂, females were not mictic, and hence, no successful mating could take place due to a lack of mating opportunities. In the combination OHJ7 ♀ × MNCHU24 ♂, 33% of females died without producing any eggs, as opposed to typically 2–9% in the other combinations (category “no eggs” in Table 3). The combination OHJ7 ♀ × MNCHU24 did not yield resting eggs, although at least three females were mictic, and male mating behavior occurred. In contrast, the reciprocal cross (MNCHU24 ♀ × OHJ7 ♂) was successful, yielding 10 resting eggs and one viable offspring.

### Inheritance of genome size

**Fig. 2:**
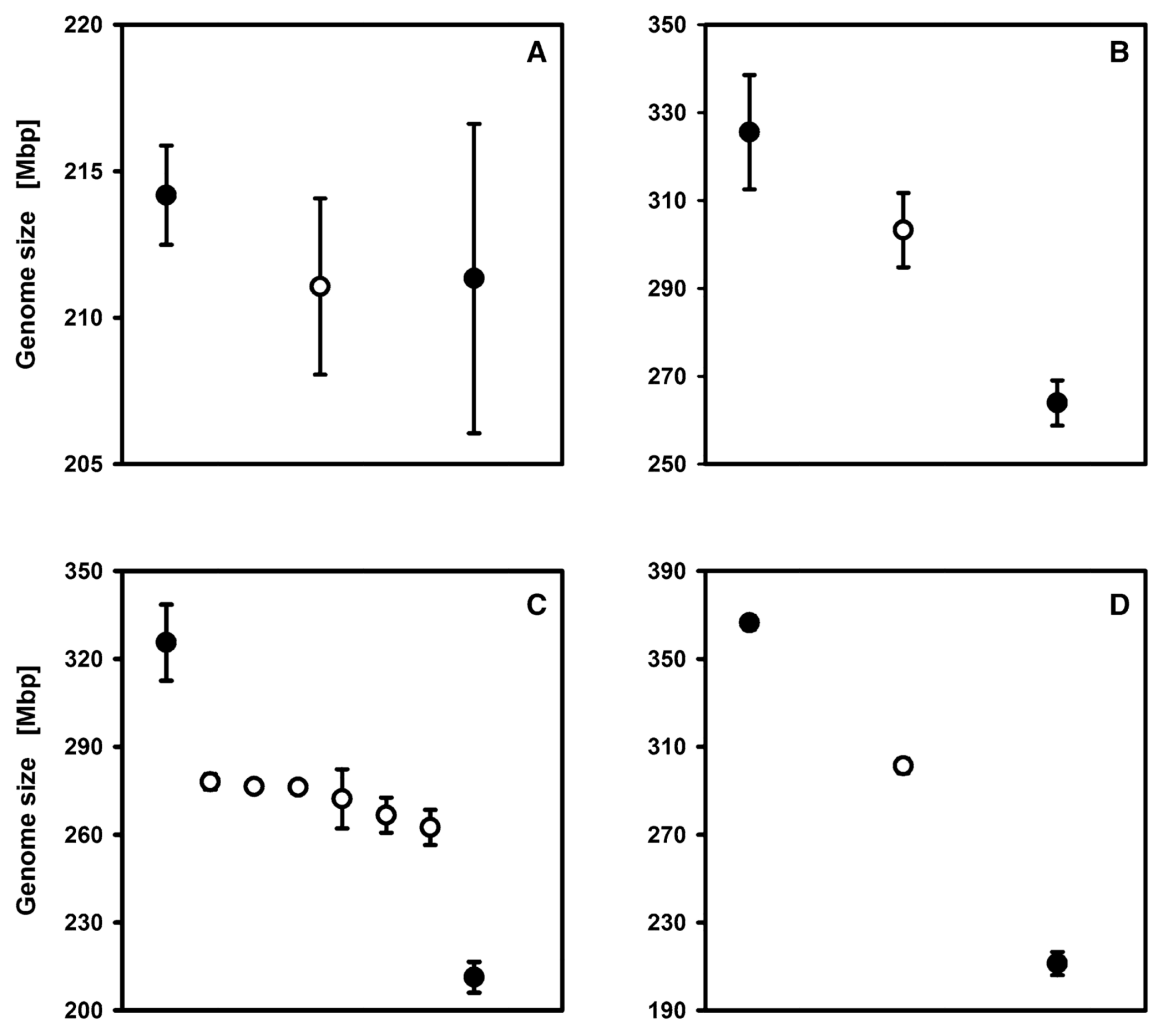
Genome sizes of parental clones and viable ‘hybrid’ offspring. **A** OHJ22 × Nakuru4, **B** OHJ7 × MNCHU24, **C** OHJ22 × MNCHU24, **D** OHJ22 × MNCHU008; *Full circles* mean parental genome sizes, *empty circles* mean offspring genome sizes

Flow cytometric measurements were performed to investigate the inheritance of genome size in ‘hybrid’ offspring, whose parents differed in genome size. Genome sizes of ‘hybrids’ were usually intermediate between their parental clones (Fig. 2). The six different ‘hybrid’ offspring that resulted from crossing the clones OHJ22 and MNCHU24 in both directions were all intermediate between their parents but varied in genome size (262–278 Mbp).

### Fitness of ‘hybrid’ offspring

**Fig. 3:**
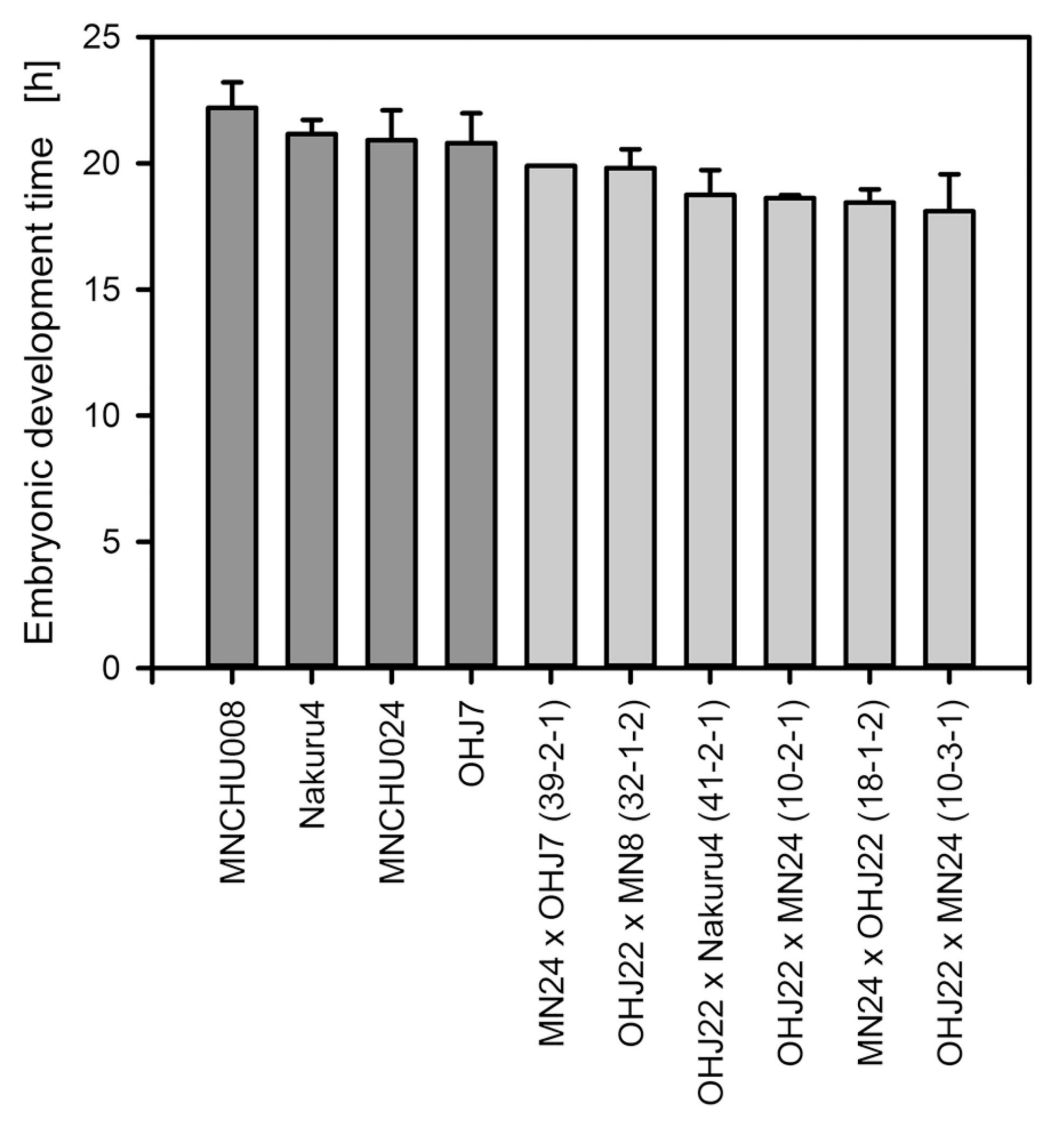
Embryonic development times of parental clones (*dark gray*) and ‘hybrid’ offspring clones (light gray). Bars represent means and standard deviations (*n* = 4-23; except MN24 × OHJ7 (39-2-1) where there was only one observation). *MN24* MNCHU24, *MN8* MNCHU008

**Fig. 4:**
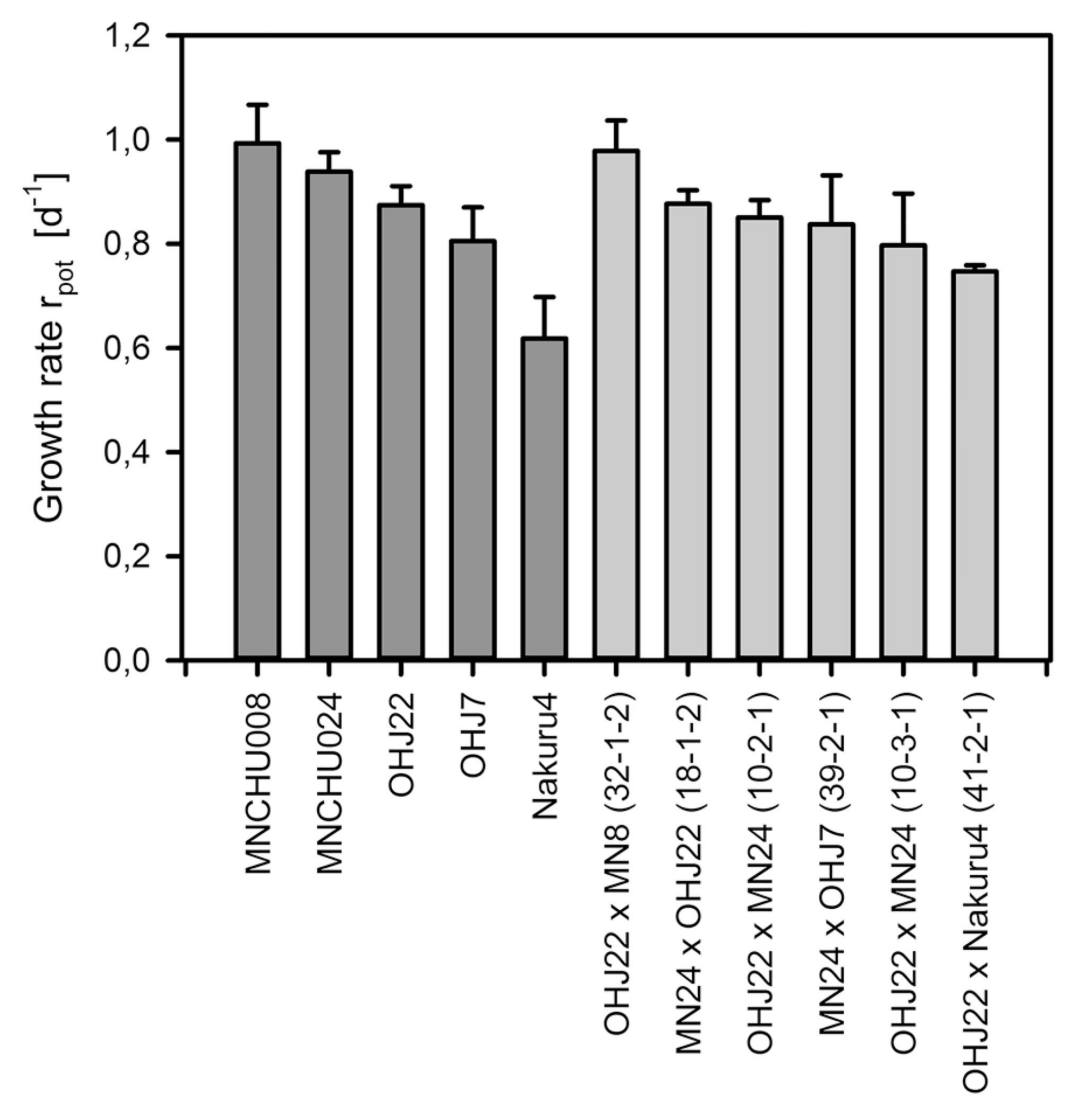
Potential growth rates rpot of parental clones (*dark gray*) and ‘hybrid’ offspring clones (*light gray*). Bars represent means and standard deviations (*n* = 3). *MN24* MNCHU24, *MN8* MNCHU008

To estimate embryonic development times, on average, eight amictic eggs per clone were measured. Embryonic development times (Fig. 3) in ‘hybrid’ offspring were significantly shorter than in their parental clones (*U* test; adjusted *H* = 6.545; df = 1; *P* = 0.011). Mean population growth rates (Fig. 4) did not differ significantly between ‘hybrid’ offspring and parents (*F* = 0.145; df = 1,12; *P* = 0.71), ranging from 0.747 days^−1^ in a OHJ22xNakuru4 ‘hybrid’ to 0.978 days^−1^ in a OHJ22xMNCHU008 ‘hybrid’ and from 0.618 d^−1^ in Nakuru4 to 0.993 d^−1^ in MNCHU008. Male production was observed in all nine ‘hybrids’.

### Amplified fragment length polymorphisms

The concatenated matrix from eight primer pairs (Supplement 1) consisted of 228 loci, and the 0:1 ratio over all samples was 1.11 ± 0.43 (mean ± standard deviation). This ratio varied in the different populations, with 0.96 ± 0.15 in OHJ, 2.08 ± 0.21 in Nakuru, and 2.13 ± 0.12 in MNCHU. ‘Hybrids’ OHJ × OHJ had a ratio of0.84 ± 0.14, OHJ × other populations 0.98 ± 0.11. The ratio was not correlated with genome size (data not shown).

Based on the DIC, the Hickory full model (*f*_α_ = 0.341932, *f*_β_ = 0.331824, $\theta_{\alpha }^{\left(\text{I}\right)}=187.914966,\theta_{\beta }^{\left(\text{I}\right)}=98.385091$) provided the best estimates for *F* statistics with the *F*_ST_-analog parameter *θ* ^(II)^ = 0.64 ± 0.03. The *f* free model (*f*_α_ = 1.006667, *f*_β_ = 1.003479, $\theta_{\alpha }^{\left(\text{I}\right)}=1004.732613,\theta_{\beta }^{\left(\text{I}\right)}=505.688296$) presented a slightly higher DIC value than the former model (2883.66 vs. 2417.76, respectively), and a *θ* ^(II)^ = 0.65 ± 0.01. Both models indicate a very strong population structure.

**Fig. 5:**
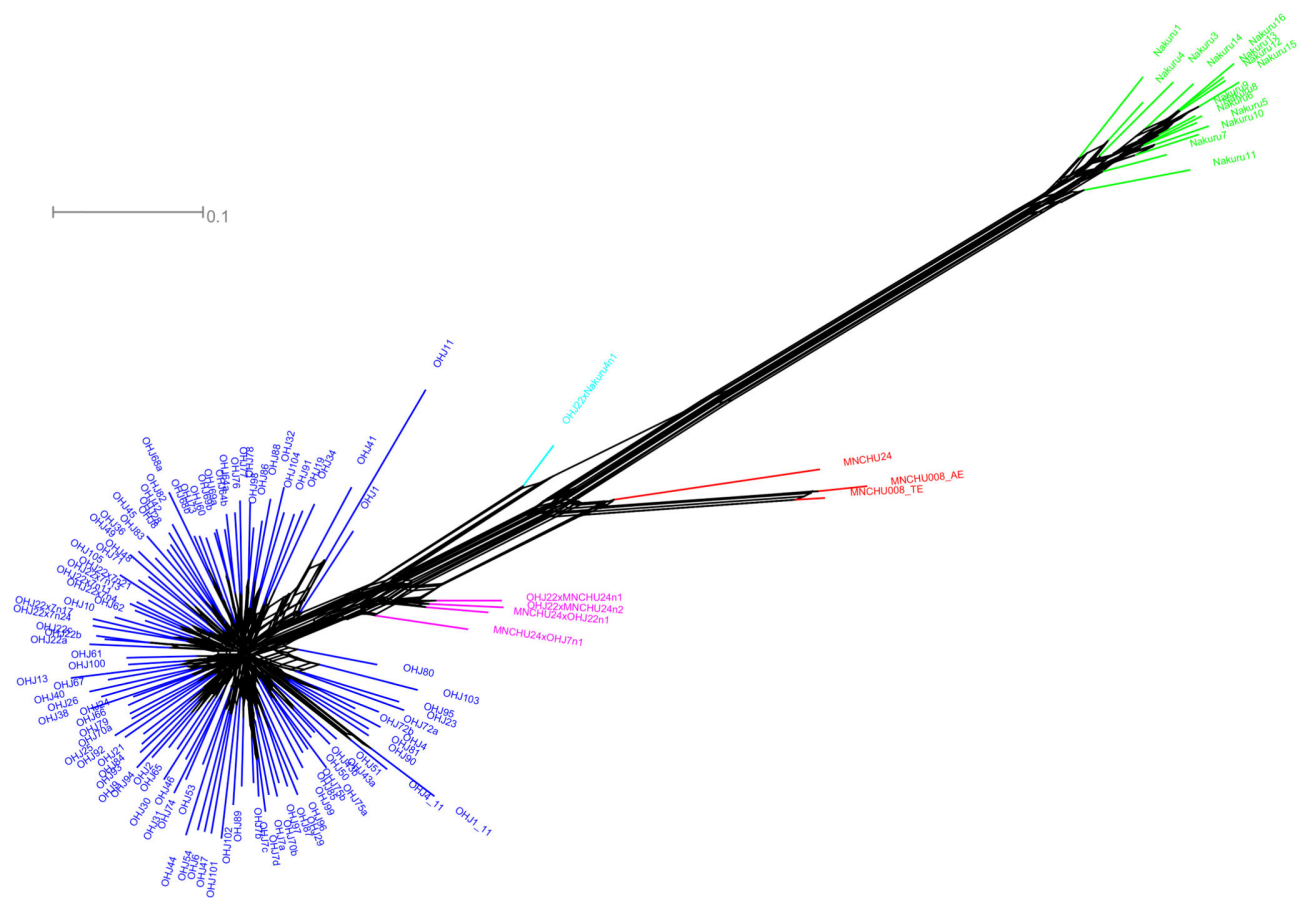
Neighbor network based on AFLP data. *Blue* OHJ, *green* Nakuru, *red* MNCHU; ‘hybrids’ appear between the main clusters: *magenta* OHJ × MNCHU, *cyan* OHJ × Nakuru

The Neighbor Network analysis (Fig. 5) conducted in SplitsTree showed two star-shaped aggregations representing OHJ and Nakuru, connected by a few long, parallel branches. From these branches, the terminal branches leading to MNCHU and the true ‘hybrids’ from the crossing experiments burgeoned out, with MNCHU almost equidistant between the both main groups and the true ‘hybrids’ close to OHJ.

**Fig. 6:**
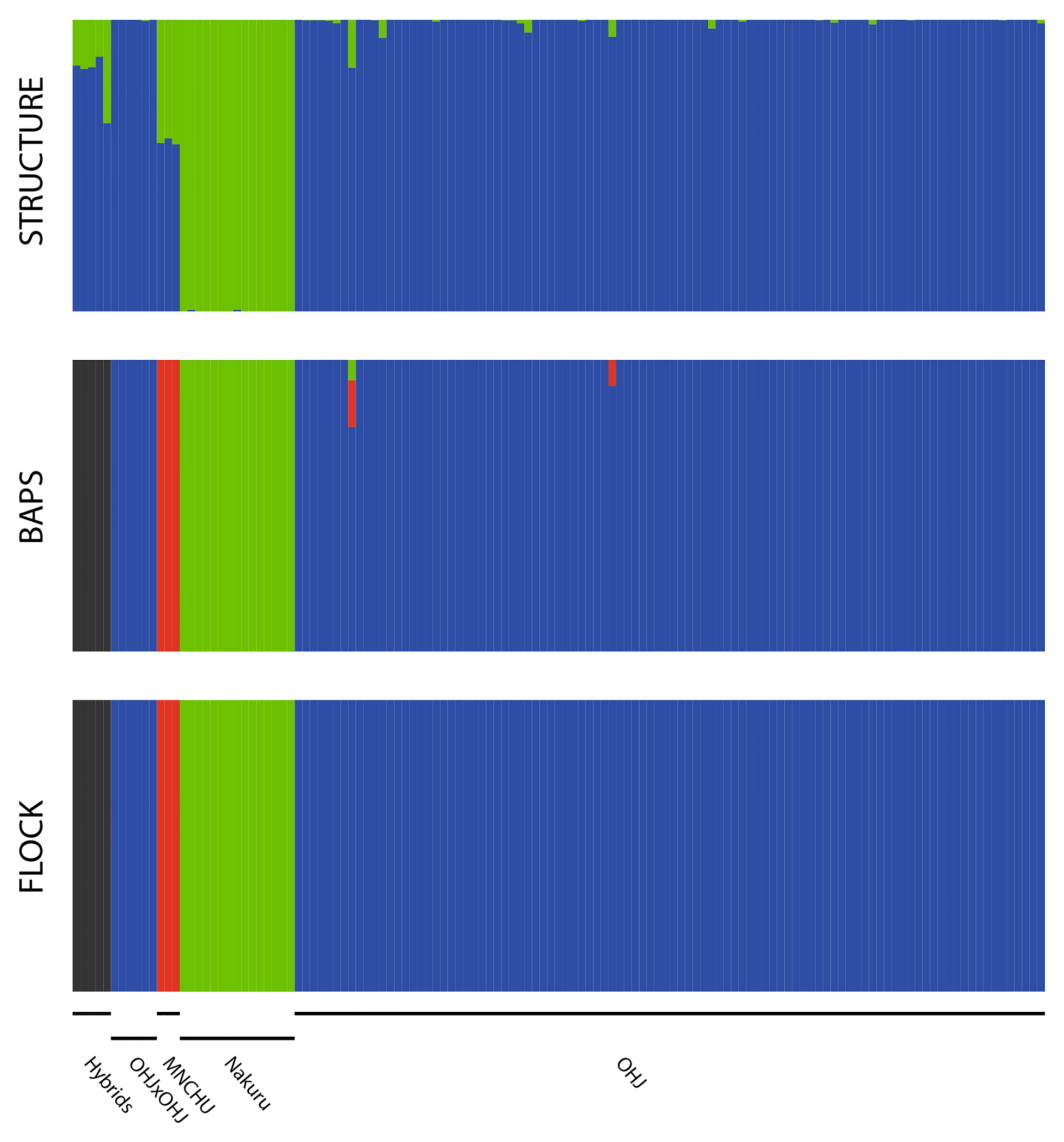
Results of three clustering algorithms applied on AFLP data using the ‘optimum’ value of K. (STRUCTURE) The Evanno method suggests two clusters, OHJ and Nakuru. The ‘hybrids’ and MNCHUare categorized as hybrids between those clusters. (BAPS) The internal algorithm of BAPS suggests four clusters: ‘Hybrids,’ OHJ, MNCHU, and Nakuru. (FLOCK) The method for finding the best estimate for *K* suggested by the authors of the software suggests four clusters. The result resembles the clustering of BAPS, with even less admixture

For the Structure results, the Evanno method suggested *K* = 2 as best estimate. Both OHJ and Nakuru appeared as almost unadmixed, separate entities (Fig. 6). MNCHU was characterized as a ca. 55:45 OHJ:Nakuru ‘hybrid,’ and also the true ‘hybrids’ from the crossing experiments showed a hybrid signature, but with higher contributions of OHJ. The clustering of individuals in BAPS revealed four clusters with 3, 5, 15, and 104 individuals, respectively, and with almost no admixture between clusters (Fig. 6). Three of these clusters represented each one of the populations OHJ, Nakuru, and MNCHU. As expected, the OHJ × OHJ crossings shared the cluster with OHJ. All interpopulation crossings were assigned to a separate, fourth cluster, with no influence whether Nakuru or MNCHU hybridized with OHJ. Signatures of admixture were negligible. Reducing the number of OHJ samples in the dataset did not change the estimates for best *K* of the Evanno and BAPS algorithms even when only six OHJ individuals remained, and thus OHJ did not represent the biggest population (Supplement 2).

The decision approach for best K in FLOCK returned K ≥ 4. Anyway, all FLOCK runs with K > 4 did not reach a plateau value, so no likelihood values for sample allocation in K > 4 were available. The results were almost identical with BAPS, with even less signal of admixture (Fig. 6).

**Fig. 7:**
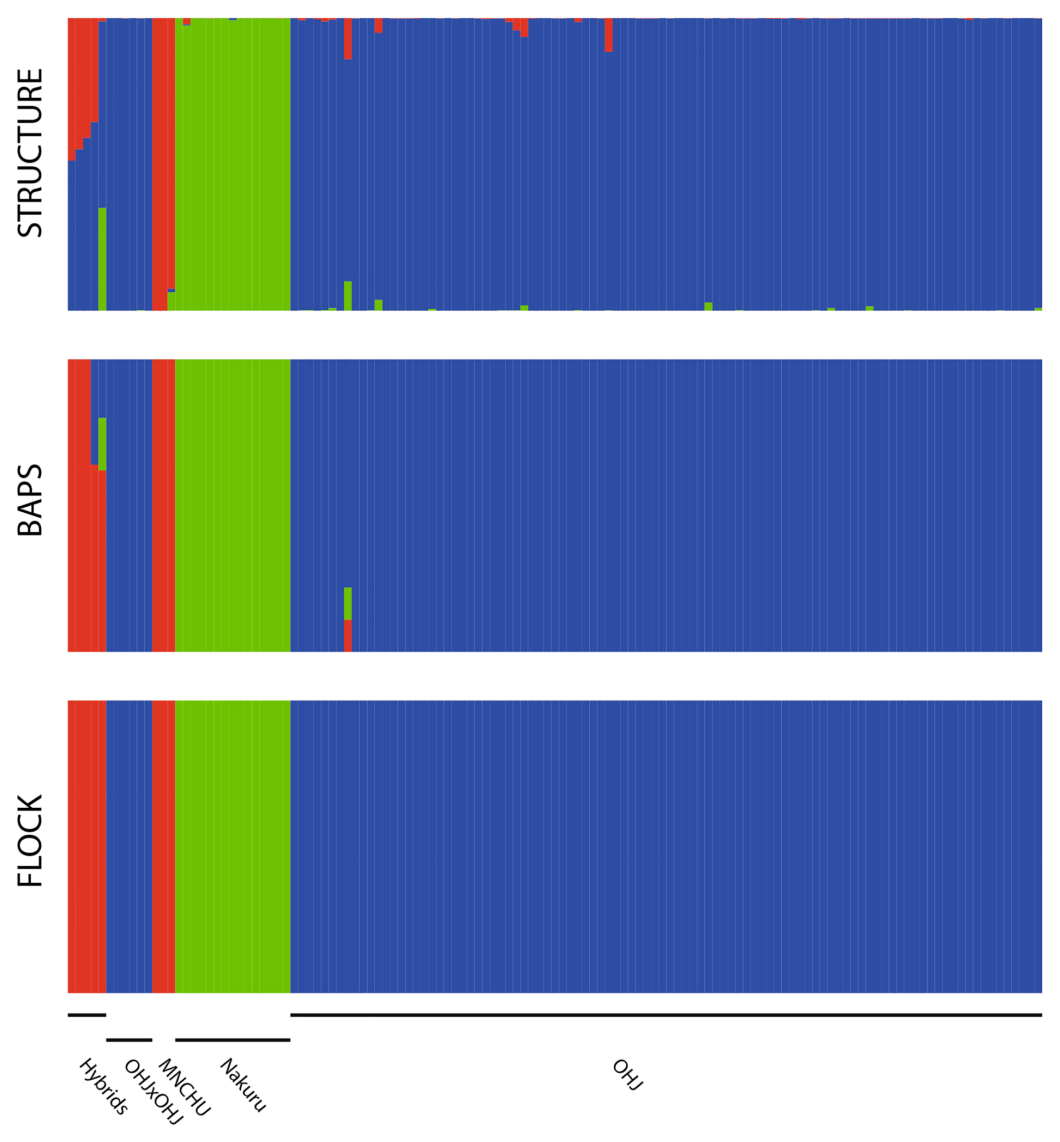
Results of three clustering algorithms applied on AFLP data when the biologically meaningful value of *K* = 3 is forced. (STRUCTURE) OHJ, MNCHU, and Nakuru form distinct clusters, with a few negligibly admixed individuals. All hybrids are correctly identified as admixed individuals with contributions of the particular parents. (BAPS) OHJ, MNCHU, and Nakuru form distinct clusters, with less signal of admixture than in STRCTURE. OHJxMNCHU hybrids are almost completely assigned to MNCHU, the OHJ × Nakuru hybrid shares fractions of all clusters, with a MNCHU majority. (FLOCK) OHJ, MNCHU, and Nakuru form distinct, unadmixed clusters. All hybrids, regardless of which parents, are identified as pure MNCHU

When ignoring the algorithms estimating the best value for K and instead visualizing the results for *K* = 3, the biologically most reasonable value given our experimental setup, all clustering programs assigned each one cluster to OHJ, Nakuru, and MNCHU. Structure correctly detected all ‘hybrids’ as admixtures of the parental clusters (Fig. 7). BAPS detected one of the four OHJ × MNCHU ‘hybrids’ as admixed and categorized the others as pure MNCHU; the OHJ × Nakuru ‘hybrid’ was seen as admixture of all three populations. FLOCK detected no admixture signals and assigned all OHJ × MNCHU and OHJ × Nakuru ‘hybrids’ to the pure MNCHU genotype.

## Discussion

### DNA sequence markers

According to their ITS1 sequences, our clones were unequivocally assigned to the same species, *B*. *asplanchnoidis*, and overall sequence diversity within populations was low despite the observed variation in nuclear DNA content. Hence, genome size differences can occur within the same species, within populations, and even within the same ITS1 and COI haplotypes in *B*. *asplanchnoidis*.

### Cross-mating experiments

The cross-mating experiments in our study were aimed at analyzing the effect of genome size differences as a potential intraspecific reproductive barrier. First, we checked for prezygotic isolation by screening for male mating behavior between clones from different populations. We observed male mating behavior in all tested combinations. Our observations suggest that no or no strong behavioral prezygotic reproductive isolation exists between *B*. *asplachnoides* clones from different populations with different genome sizes. Intraspecific behavioral prezygotic reproductive isolation in the *B*. *plicatilis* species complex has been tested previously (Suatoni et al., 2006; Berrieman et al., 2005), but these studies only used species, which according to current knowledge seem not to possess considerable intraspecific variation in genome size. Suatoni et al. (2006) detected no prezygotic reproductive isolation in any intraspecific matings; in contrast, in the same study, eight of 14 interspecific crosses failed already at the mate recognition stage. To our knowledge, only Berrieman et al. (2005) described some degree of intraspecific behavioral isolation between two *B*. *plicatilis* strains. Gribble & Mark Welch (2012) assessed male mating behavior quantitatively in reciprocal mating assays within and between conspecific isolates of the *B*. *plicatilis* complex and found differences in the rates of circling and copulation, i.e., rates of copulation were typically highest in self-crosses. More subtle quantitative differences in male mating behavior toward females with similar or more different genome sizes would not have been detected by our experimental design, but could be of relevance in nature.

Successful resting egg production and hatching in the cross-mating experiments showed that mictic *B*. *asplanchnoidis* females can in principle be fertilized by males from other populations with different genome sizes. Thus, we can conclude that there areo at least no absolute postzygotic reproductive barriers. However, more subtle reductions in fertilization success would not have been detected in our experiments, such as decreased fertilization rates or increased embryo mortality.

An unexpected observation was that variable proportions of females died without producing eggs (category “no eggs” in Table 3), especially in the combination OHJ7 ♀ × MNCHU24 ♂. We suggest two possible explanations for this observation. First, these females were mictic females, which were fertilized but could not produce resting eggs due to postzygotic reproductive barriers. Second, these females might have been of any reproductive type (amictic or mictic) and were simply harmed by too many intrusive male mating attempts (and thus died without producing any eggs). Fertilization in brachionids takes place by traumatic hypodermic insemination (Gilbert, 1988). Snell et al. (2007) mentioned that insertion of the penis through the coronal membrane might cause loss of pseudocoelomic fluid or facilitate pathogen infections. We hypothesize that females which die early (shortly after matings) might be likely caused by injuries, while ‘unreproductive’ females with normal life spans could be mictic females that mated, but could not successfully produce resting eggs due to reproductive barriers. We observed both in our experiments: ‘unreproductive’ females with short and normal lifespans, but we did not count them in separate categories. In future studies, larger sample sizes and control treatments without males (to estimate the ratios of mictic females in populations) might help to infer the likely reproductive type of ‘undetermined’ females, while mating tests with different male to female ratios could help to test whether ratios above a certain threshold are correlated with higher percentages of ‘unreproductive’ females.

As a side result, we found that hatching rates of male eggs were surprisingly low in the two Mongolian clones, much lower than in the OHJ and Nakuru clones, respectively (Table 4). An explanation could be that a recessive and partially lethal allele is present in the two MNCHU clones, which exerts its full negative effect only in the haploid males. Since those clones have been cultivated asexually in the lab for several years, they might have accumulated deleterious mutations. In fact, larger genomes are suspected to be more prone to accumulate deleterious mutations (Lynch, 2007, 2010).

### Inheritance of genome size

Genome sizes of ‘hybrid’ offspring were intermediate between their parental clones (see Fig. 2). This result is in accordance with inheritance patterns of genome size described for other organisms. For instance, Šmarda et al. (2007; 2008; 2009) studied the inheritance of genome size in the grass *Festuca pallens*, which also exhibits intraspecific and intrapopulation genome size variation, and found that in reciprocal crosses of diploid plants, progeny genome size ranged between the parental sizes. However, also other inheritance patterns have been described. For example, Rayburn et al. (1993) showed that some parental combinations of maize could produce F1 ‘hybrids’ with larger genome sizes than their respective parental means. At any rate, our finding of intermediate genome sizes further corroborates the notion that our ‘hybrid’ clones were in fact ‘hybrids’ and therefore that the cross-mating experiments were successful.

The underlying cause of genome size variation in *B*. *asplanchnoidis* is still unknown. In general, genome size variation across species can result from gene-, chromosome-, or genome duplications, from variations in the length of introns, number of transposons, and the amount of single repetitive DNA (Cavalier-Smith, 2005; Gregory, 2005; Lynch, 2007). Twomodels seem possible in the case of *B*. *asplanchnoidis*: individuals could differ in chromosome number, e.g., by having different numbers of (supernumerary) chromosomes, or individuals could differ in chromosome size of homeologous (=partially homologous) chromosomes (e.g., Šmarda et al., 2008; Jeffery et al., 2016). At present, we cannot distinguish between these two possibilities as we still lack karyological data on *B*. *asplanchnoidis*. However, the second model (differentially sized homeologous chromosomes) seems to be more plausible since our crossing experiments indicate that large genome size differences do not interfere with meiotic processes (e.g., chromosome pairing) and recombination.

### Fitness of ‘hybrid’ offspring

Postzygotic isolation mechanisms may prevent hybrids from passing on their genes, e.g., through hybrid inviability, hybrid sterility, or hybrid breakdown. Postzygotic mechanisms have been suggested as a likely mechanism for reproductive isolation between the more distantly related clades of the *B*. *plicatilis* complex (Rumengan et al., 1991; Fu et al., 1993). In our study, we tested for postzygotic reproductive barriers affecting the fitness of ‘hybrid’ offspring.

Our results suggest that there is no or at least no strong postzygotic isolation among *B*. *asplanchnoidis* clones and populations that differ in genome size. Our inference is based on four observations: First, we obtained viable and fertile ‘hybrid’ offspring in the majority of crossings (Table 3), which demonstrates that there is at least no absolute postzygotic reproductive isolation. However, since not all resting eggs hatched, the possibility of quantitative effects should be taken into account in future studies. Second, there were also no significant differences in growth rate between ‘hybrid’ offspring and their parents. Third, embryonic development times were significantly shorter in the ‘hybrid’ offspring, suggesting that they did not suffer from outbreeding depression but rather enjoyed some hybrid vigor in this life history trait. Fourth, male production was observed in all nine ‘hybrid’ clones, which indicates that meiosis is undisturbed (this can be concluded because males are haploid in this species). Taken together, our results suggest that members of different populations with differing genome size (up to 1.7-fold) interbreed successfully, at least under our noncompetitive laboratory conditions, and produce offspring of equal or higher fitness. Therefore, genome size differences within this species do not necessarily lead to reproductive isolation.

### Amplified fragment length polymorphisms

Amplified fragment length polymorphisms (AFLP) have been successfully used in many population genetic analyses (Bensch & Akesson, 2005). Especially in polyploid organisms, where codominant markers like microsatellites are not applicable, AFLP serves as a powerful alternative (Meudt & Clarke, 2007). Relevant for our work, such studies have proven the suitability of AFLP for organisms with different genome sizes (Kardolus et al., 1998). Genome size, on the other hand, directly influences the number of peaks observed in AFLP profiles. In plants, a genome size range from 294 to 8260 MB has been identified as suitable for the standard AFLP approach with three selective nucleotides in the second amplification step (Fay et al., 2005). The genomes analyzed here (211–326 MB) are all at the lower boundary of this range, and indeed the number of scoreable loci per primer set (average = 57.0 loci) was lower than for instance in basal insects (116.5 loci; Dejaco et al., 2016) or ants (135 loci, genus *Tetramorium*; Arthofer et al., unpublished).

Our population genetic analyses on the AFLP data indicated a strong population structure among the rotifer clones of our study and were able, in accordance with their geographical separation, to distinguish the main populations OHJ, Nakuru, and MNCHU. Nevertheless, a closer examination of the results reveals some interesting and partially contradictory results.

Under the unified species concept (de Queiroz, 2007), using as species-delimitation criteria genotypic clustering (Mallet, 1995) and reproductive incompatibility (Mayr, 1942), OHJ, Nakuru, and MNCHU should represent three separate but still hybridizing species. We would thus expect the true number of clusters in our population sample (*K*) to be 3. However, the Evanno method applied on the Structure data suggested *K* = 2, while BAPS and FLOCK suggested *K* = 4. We interpret the BAPS and FLOCK results as oversplitting, as at least BAPS has been repeatedly shown to detect substructures at fine scale for a range of organisms (Latch et al., 2006; Arthofer et al., 2013). On the other hand, Structure is known to generate misleading results (e.g., putting dissimilar samples into the same cluster or generating hybrid individuals which are in fact pure ones) in case of highly uneven population sizes (Kalinowski, 2011; and own results, unpublished). In our study, OHJ samples were highly overrepresented, and thus we repeated the analyses using subsamples with reduced numbers of OHJ. However, even the most reduced datasets suggested a value of *K* = 2. These results may indicate that MNCHU is not a well-separated population, but indeed rather a ‘hybrid’ between OHJ and Nakuru, in line with the results from Splitstree, where the MNCHU samples are located almost equidistant on a long branch between the OHJ and Nakuru populations. More analyses, involving additional clones from the MNCHU population and other *B*. *asplanchniodis* populations, will be needed to solve this question. After all, finding the ‘true’ value for K remains an important issue in molecular ecology (Evanno et al., 2005; Kalinowski, 2011; Duchesne & Turgeon, 2012).

Despite the unclear status of MNCHU, true ‘hybrids’ between the three populations were produced under laboratory conditions and assessed by AFLP. Only the Structure approach with *K* = 3 (which, however was not suggested by the Evanno-procedure) correctly identified the ‘hybrids’ as admixed individuals and assigned them to the correct parents. BAPS failed partially, and FLOCK completely in this task, indicating that the bioinformatic methods for hybrid detection, at least in this case, are still far from being optimal.

All algorithms applied in this study characterized the OHJ population as one homogeneous population with negligible internal structure. Thus, there is currently no indication for reduction of gene flow among members of the OHJ population, despite the observed variation in genome size (typical range 205–270 Mbp). Altogether, this suggests that intrapopulation genome size variation did not lead to assortative mating or any other reproductive barriers, which is in agreement with our results from the interpopulation crosses.

## Conclusions

We did not find evidence supporting the hypothesis that speciation processes are currently occurring in *B*. *asplanchnoidis*. Different populations of *B*. *asplanchnoidis*, while geographically isolated, can still interbreed in the laboratory. Furthermore, we detected signs of natural interbreeding, as the analysis of AFLP data suggested that the clones from the Mongolian population (MNCHU) might be ‘hybrids’ between the Austrian (OHJ) and East African populations (Nakuru). In conclusion, the enormous intraspecific genome size differences within *B*. *asplanchnoidis* do not seem to cause reproductive isolation or reduced fitness in ‘hybrid’ offspring. Future experiments, which might address subtle quantitative negative effects (e.g., reduced resting egg hatching rates), are needed to completely rule out that genome size differences play any role in reproductive isolation within this group.

## Supplementary Material

The online version of this article (doi:10.1007/s10750-016-2872-x) contains supplementary material, which is available to authorized users.

Supplement 1

Supplement 2

## Figures and Tables

**Table 1:** Clones and genome size range

Population	Clones		Genome size (Mbp)
	# Clones	Nomenclature	
OHJ	86	OHJ1-105	230–256^a^
			205–270^b^
Nakuru	15	Nakuru1-16	217–222^a^
MNCHU	2	MNCHU24, MNCHU008	340–407^a^

*Mbp* million base pairs

a Stelzer et al. (2011)

b Stelzer et al. (in preparation)

**Table 2:** Genome size differences of parental clones

Clone	GS (Mbp)	Source
OHJ22	211	Stelzer et al. (2011) + this study
Nakuru4	214	this study
OHJ7	264	Stelzer et al. (2011) + this study
MNCHU24	326	Stelzer et al. (2011) + this study
MNCHU008	366	Stelzer et al. (2011) + this study

*GS* genome size; *Mbp* million base pairs

**Table 3:** Summary of the cross-mating experiment

Parental clones	GS difference (Mbp)	Mating behavior	Fertilization of mictic ♀				Resting eggs		Offspring
			# Total in	# No eggs	# Mictic	# Fertilized	# Produced	# Hatched	# Fertile
OHJ22 ♀ × Nakuru4 ♂	3	Yes	97	2	2	2	2	1	1
OHJ7 ♀ × MNCHU24 ♂	62	Yes	139	46	3	0			
MNCHU24 ♀ × OHJ7 ♂	62	Yes	223	17	4	4	10	1	1
OHJ22 ♀ × MNCHU24 ♂	115	Yes	323	29	34	5	7	5	5
MNCHU24 ♀ × OHJ22 ♂	115	Yes	671	62	7	5	11	2	1
OHJ22 ♀ × MNCHU008 ♂	155	Yes	98	5	9	1	2	1	1
MNCHU008 ♀ × OHJ22 ♂	155	Yes	83	3	0				

*GS* genome size, *Mbp* million base pairs; *in* individuals

**Table 4:** Hatching rates of female and male eggs of parental clones

Clone	♀ eggs		♂ eggs	
	#	% hatched	#	% hatched
Nakuru4	n.a.*	n.a.	16	100
OHJ22	542	97	378	97
OHJ7	148	95	108	91
MNCHU24	1040	96	566	9
MNCHU008	202	89	61	3

* Hatching rates of Nakuru4 female eggs were not tested
