# Supplementary material for: Do genome size differences within Brachionus asplanchnoidis (Rotifera, Monogononta) cause reproductive barriers among geographic populations?
Source: Hydrobiologia. Author manuscript; Available in PMC 2021 Nov 10. (PMC7611973; doi:10.1007/s10750-016-2872-x)
Supplement: Supplement 2 [file EMS137972-supplement-Supplement_2.zip › 73del_r4/73del_r4.tar.gz!/summary.html]

Structure Harvester


Structure Harvester

- Home
- News
- FAQ
- Contact

# Job afternoon-sunset-87c0

This output file was generated at:

2015-Dec-07 05:43:05 PST

This document is not permanent. It will automatically be removed from the server in seven (7) days. Please save or print it for your records. If images are missing, try reloading; this sometimes happens under heavy server load.

---

Single file archive including this page, all images, all clumpp files: download. [.tar.gz]

## L(K)

  

L(K): pdf   eps

---

## Clumpp files

K = 1 Clumpp indfile    K = 1 Clumpp popfile

K = 2 Clumpp indfile    K = 2 Clumpp popfile

K = 3 Clumpp indfile    K = 3 Clumpp popfile

K = 4 Clumpp indfile    K = 4 Clumpp popfile

K = 5 Clumpp indfile    K = 5 Clumpp popfile

K = 6 Clumpp indfile    K = 6 Clumpp popfile

K = 7 Clumpp indfile    K = 7 Clumpp popfile

---

## Evanno method

\*Evanno et al., 2005. Molecular Ecology 14, 2611 - 2620. How are we calculating this? Look at the FAQ.

  

L'(K): pdf   eps

---

  

|L''(K)|: pdf   eps

---

  

Delta K: pdf   eps

---

The Evanno table output is also available as a tab-delimited text file (for use with Excel) here.

| K | Reps | Mean LnP(K) | Stdev LnP(K) | Ln'(K) | \|Ln''(K)\| | Delta K |
| --- | --- | --- | --- | --- | --- | --- |
|  |  |  |  |  |  |  |
| --- | --- | --- | --- | --- | --- | --- |
| 1 | 10 | -14425.710000 | 1.347797 | — | — | — |
| 2 | 10 | -7138.650000 | 0.408928 | 7287.060000 | 6509.380000 | 15918.151361 |
| 3 | 10 | -6360.970000 | 2.092871 | 777.680000 | 586.050000 | 280.021970 |
| 4 | 10 | -6169.340000 | 597.852881 | 191.630000 | 250.810000 | 0.419518 |
| 5 | 10 | -5726.900000 | 103.653461 | 442.440000 | 565.510000 | 5.455775 |
| 6 | 10 | -5849.970000 | 963.203507 | -123.070000 | 550.270000 | 0.571292 |
| 7 | 10 | -5422.770000 | 353.961200 | 427.200000 | — | — |

---

## Raw STRUCTURE output

The raw STRUCTURE output is also available as a tab-delimited text file (for use with Excel) here.

| File name | Run # | K | Est. Ln prob. of data | Mean value of Ln likelihood | Variance of Ln likelihood |
| --- | --- | --- | --- | --- | --- |
| 73del\_r4\_run\_8\_f | 8 | 1 | -14425.0 | -14369.5 | 111.1 |
| 73del\_r4\_run\_7\_f | 7 | 1 | -14425.1 | -14369.3 | 111.5 |
| 73del\_r4\_run\_5\_f | 5 | 1 | -14426.3 | -14369.5 | 113.6 |
| 73del\_r4\_run\_6\_f | 6 | 1 | -14424.4 | -14369.2 | 110.4 |
| 73del\_r4\_run\_2\_f | 2 | 1 | -14425.9 | -14369.4 | 113.1 |
| 73del\_r4\_run\_1\_f | 1 | 1 | -14424.4 | -14369.3 | 110.1 |
| 73del\_r4\_run\_10\_f | 10 | 1 | -14427.8 | -14369.6 | 116.5 |
| 73del\_r4\_run\_3\_f | 3 | 1 | -14425.2 | -14369.4 | 111.6 |
| 73del\_r4\_run\_9\_f | 9 | 1 | -14424.8 | -14369.4 | 110.7 |
| 73del\_r4\_run\_4\_f | 4 | 1 | -14428.2 | -14369.6 | 117.3 |
| 73del\_r4\_run\_13\_f | 13 | 2 | -7139.0 | -7059.9 | 158.2 |
| 73del\_r4\_run\_18\_f | 18 | 2 | -7138.4 | -7059.7 | 157.3 |
| 73del\_r4\_run\_14\_f | 14 | 2 | -7138.9 | -7059.9 | 158.1 |
| 73del\_r4\_run\_15\_f | 15 | 2 | -7138.0 | -7059.7 | 156.5 |
| 73del\_r4\_run\_16\_f | 16 | 2 | -7139.2 | -7059.9 | 158.6 |
| 73del\_r4\_run\_12\_f | 12 | 2 | -7138.2 | -7059.9 | 156.7 |
| 73del\_r4\_run\_20\_f | 20 | 2 | -7138.7 | -7059.9 | 157.6 |
| 73del\_r4\_run\_17\_f | 17 | 2 | -7139.1 | -7060.0 | 158.3 |
| 73del\_r4\_run\_11\_f | 11 | 2 | -7138.3 | -7059.7 | 157.1 |
| 73del\_r4\_run\_19\_f | 19 | 2 | -7138.7 | -7059.7 | 158.0 |
| 73del\_r4\_run\_26\_f | 26 | 3 | -6362.2 | -6237.1 | 250.2 |
| 73del\_r4\_run\_24\_f | 24 | 3 | -6363.6 | -6237.5 | 252.3 |
| 73del\_r4\_run\_25\_f | 25 | 3 | -6361.3 | -6236.4 | 249.8 |
| 73del\_r4\_run\_29\_f | 29 | 3 | -6359.2 | -6236.5 | 245.5 |
| 73del\_r4\_run\_22\_f | 22 | 3 | -6363.7 | -6236.9 | 253.6 |
| 73del\_r4\_run\_21\_f | 21 | 3 | -6357.8 | -6236.5 | 242.7 |
| 73del\_r4\_run\_28\_f | 28 | 3 | -6362.3 | -6236.7 | 251.1 |
| 73del\_r4\_run\_30\_f | 30 | 3 | -6358.6 | -6235.9 | 245.3 |
| 73del\_r4\_run\_27\_f | 27 | 3 | -6361.6 | -6236.6 | 250.1 |
| 73del\_r4\_run\_23\_f | 23 | 3 | -6359.4 | -6236.7 | 245.3 |
| 73del\_r4\_run\_32\_f | 32 | 4 | -6128.1 | -5969.0 | 318.2 |
| 73del\_r4\_run\_37\_f | 37 | 4 | -5946.5 | -5824.6 | 243.7 |
| 73del\_r4\_run\_35\_f | 35 | 4 | -5944.7 | -5824.2 | 240.9 |
| 73del\_r4\_run\_38\_f | 38 | 4 | -5944.6 | -5824.2 | 240.9 |
| 73del\_r4\_run\_31\_f | 31 | 4 | -5946.6 | -5824.3 | 244.6 |
| 73del\_r4\_run\_36\_f | 36 | 4 | -7860.0 | -5854.6 | 4010.7 |
| 73del\_r4\_run\_40\_f | 40 | 4 | -5946.4 | -5824.4 | 243.9 |
| 73del\_r4\_run\_39\_f | 39 | 4 | -5946.3 | -5824.6 | 243.3 |
| 73del\_r4\_run\_33\_f | 33 | 4 | -6084.2 | -5972.1 | 224.3 |
| 73del\_r4\_run\_34\_f | 34 | 4 | -5946.0 | -5824.1 | 244.0 |
| 73del\_r4\_run\_50\_f | 50 | 5 | -5663.3 | -5543.1 | 240.5 |
| 73del\_r4\_run\_46\_f | 46 | 5 | -5797.0 | -5689.0 | 215.9 |
| 73del\_r4\_run\_44\_f | 44 | 5 | -5664.5 | -5543.2 | 242.4 |
| 73del\_r4\_run\_47\_f | 47 | 5 | -5666.9 | -5544.1 | 245.7 |
| 73del\_r4\_run\_41\_f | 41 | 5 | -5663.6 | -5543.3 | 240.5 |
| 73del\_r4\_run\_48\_f | 48 | 5 | -5663.5 | -5543.1 | 240.9 |
| 73del\_r4\_run\_45\_f | 45 | 5 | -5708.1 | -5586.7 | 242.8 |
| 73del\_r4\_run\_43\_f | 43 | 5 | -5977.8 | -5551.6 | 852.4 |
| 73del\_r4\_run\_42\_f | 42 | 5 | -5798.7 | -5690.1 | 217.1 |
| 73del\_r4\_run\_49\_f | 49 | 5 | -5665.6 | -5543.9 | 243.4 |
| 73del\_r4\_run\_60\_f | 60 | 6 | -5473.0 | -5350.1 | 245.7 |
| 73del\_r4\_run\_54\_f | 54 | 6 | -5513.5 | -5349.8 | 327.6 |
| 73del\_r4\_run\_52\_f | 52 | 6 | -5430.5 | -5306.1 | 248.9 |
| 73del\_r4\_run\_58\_f | 58 | 6 | -5429.8 | -5306.2 | 247.2 |
| 73del\_r4\_run\_57\_f | 57 | 6 | -5473.2 | -5350.1 | 246.3 |
| 73del\_r4\_run\_56\_f | 56 | 6 | -5428.7 | -5305.6 | 246.3 |
| 73del\_r4\_run\_51\_f | 51 | 6 | -5430.3 | -5306.3 | 247.9 |
| 73del\_r4\_run\_55\_f | 55 | 6 | -5474.1 | -5350.0 | 248.2 |
| 73del\_r4\_run\_59\_f | 59 | 6 | -8461.2 | -5413.9 | 6094.6 |
| 73del\_r4\_run\_53\_f | 53 | 6 | -6385.4 | -5378.9 | 2012.9 |
| 73del\_r4\_run\_61\_f | 61 | 7 | -5310.5 | -5175.7 | 269.5 |
| 73del\_r4\_run\_63\_f | 63 | 7 | -5240.3 | -5113.9 | 252.6 |
| 73del\_r4\_run\_69\_f | 69 | 7 | -5475.7 | -5103.1 | 745.1 |
| 73del\_r4\_run\_67\_f | 67 | 7 | -5241.3 | -5113.7 | 255.1 |
| 73del\_r4\_run\_68\_f | 68 | 7 | -5316.2 | -5175.1 | 282.0 |
| 73del\_r4\_run\_66\_f | 66 | 7 | -5432.2 | -5155.3 | 554.0 |
| 73del\_r4\_run\_62\_f | 62 | 7 | -6403.1 | -5123.1 | 2560.1 |
| 73del\_r4\_run\_70\_f | 70 | 7 | -5241.1 | -5113.5 | 255.2 |
| 73del\_r4\_run\_64\_f | 64 | 7 | -5254.0 | -5125.6 | 256.8 |
| 73del\_r4\_run\_65\_f | 65 | 7 | -5313.3 | -5173.5 | 279.5 |

---

## CITATION

```
Earl, Dent A. and vonHoldt, Bridgett M. (2012)
STRUCTURE HARVESTER: a website and program for visualizing 
STRUCTURE output and implementing the Evanno method. 
Conservation Genetics Resources vol. 4 (2) pp. 359-361 doi: 10.1007/s12686-011-9548-7
Core version: vA.2 July 2014
Plot version: vA.1 November 2012
Web version: v0.6.94 July 2014
```

---


© Dent Earl 2007-2014
