# Supplementary material for: Do genome size differences within Brachionus asplanchnoidis (Rotifera, Monogononta) cause reproductive barriers among geographic populations?
Source: Hydrobiologia. Author manuscript; Available in PMC 2021 Nov 10. (PMC7611973; doi:10.1007/s10750-016-2872-x)
Supplement: Supplement 2 [file EMS137972-supplement-Supplement_2.zip › 49del_r2/49del_r2.tar.gz!/meanLnProb.pdf]

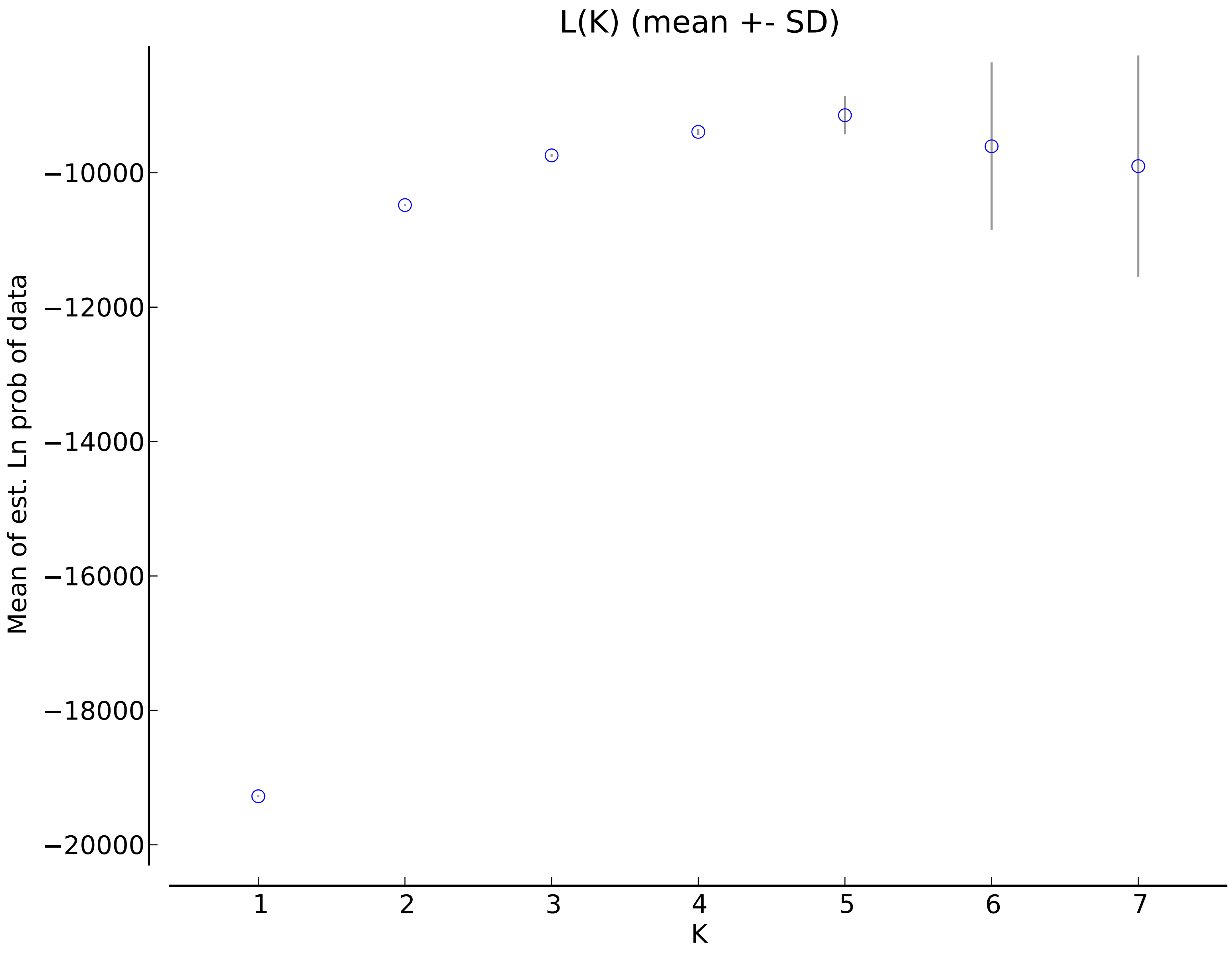
L(K) (mean +- SD)
Mean of est. Ln prob of data
−10000
−12000
−14000
−16000
−18000
−20000
1
2
3
4
5
6
7
K
