# Supplementary material for: Do genome size differences within Brachionus asplanchnoidis (Rotifera, Monogononta) cause reproductive barriers among geographic populations?
Source: Hydrobiologia. Author manuscript; Available in PMC 2021 Nov 10. (PMC7611973; doi:10.1007/s10750-016-2872-x)
Supplement: Supplement 2 [file EMS137972-supplement-Supplement_2.zip › 73del_r1/73del_r1.tar.gz!/deltaK.pdf]

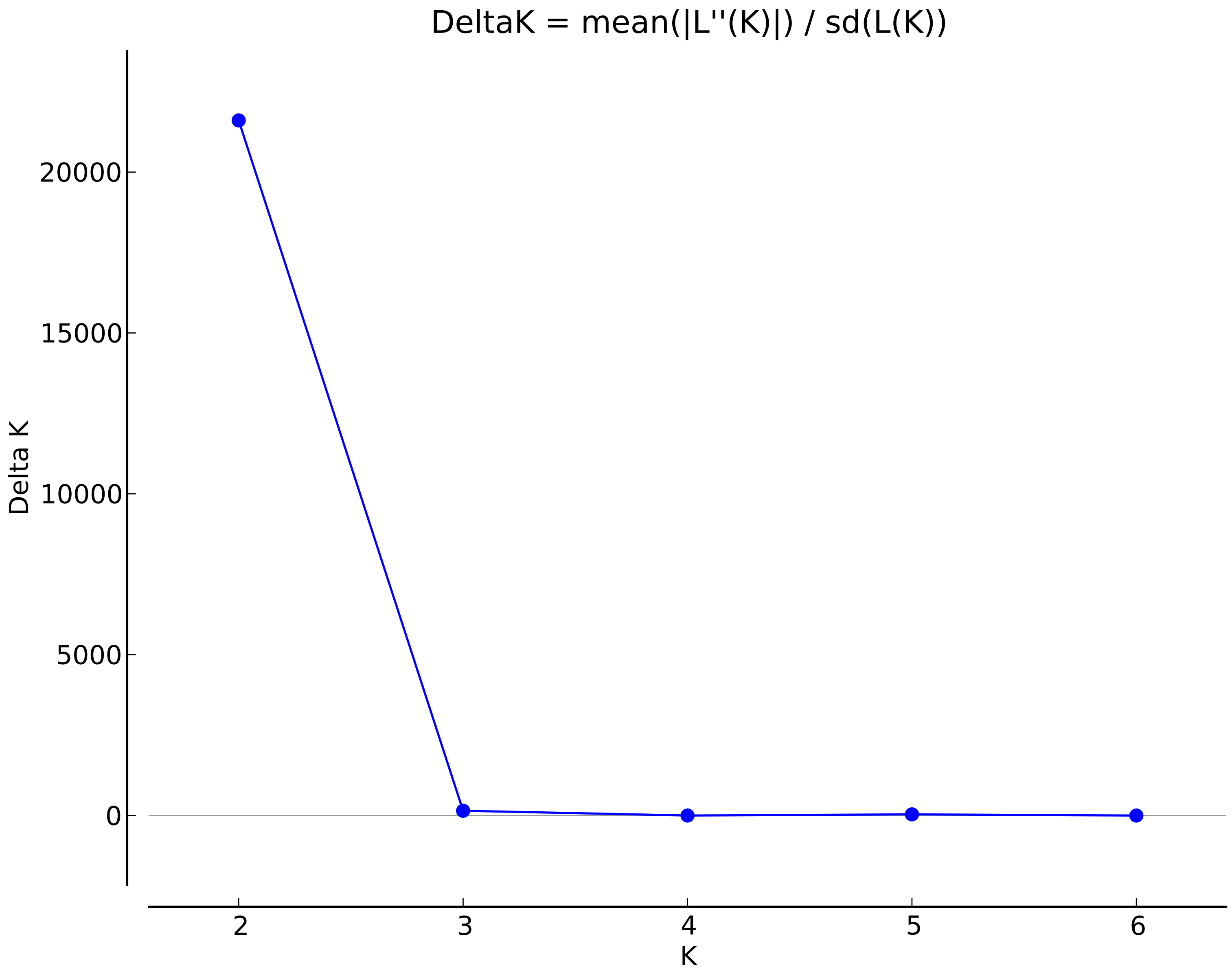
DeltaK = mean(|L''(K)|) / sd(L(K))
Delta K
20000
15000
10000
5000
0
2
3
4
5
6
K
