# Supplementary material for: Do genome size differences within Brachionus asplanchnoidis (Rotifera, Monogononta) cause reproductive barriers among geographic populations?
Source: Hydrobiologia. Author manuscript; Available in PMC 2021 Nov 10. (PMC7611973; doi:10.1007/s10750-016-2872-x)
Supplement: Supplement 2 [file EMS137972-supplement-Supplement_2.zip › 86del_r2/86del_r2.tar.gz!/lnPPK.pdf]

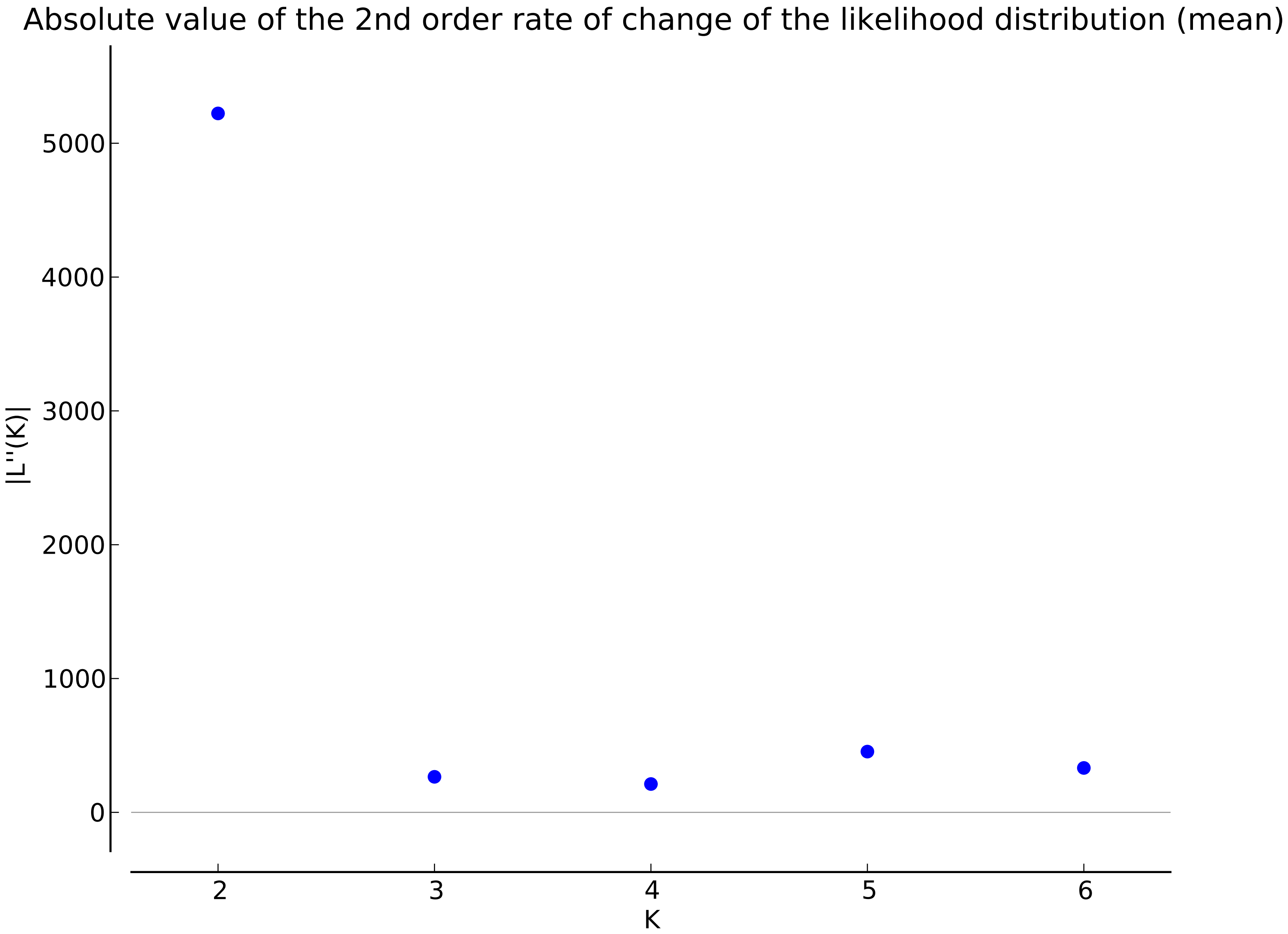

Absolute value of the 2nd order rate of change of the likelihood distribution (mean)
|L''(K)|
5000
4000
3000
2000
1000
0
2
3
4
5
6
K
