# Supplementary material for: Do genome size differences within Brachionus asplanchnoidis (Rotifera, Monogononta) cause reproductive barriers among geographic populations?
Source: Hydrobiologia. Author manuscript; Available in PMC 2021 Nov 10. (PMC7611973; doi:10.1007/s10750-016-2872-x)
Supplement: Supplement 2 [file EMS137972-supplement-Supplement_2.zip › 92del_r1/92del_r1.tar.gz!/meanLnProb.pdf]

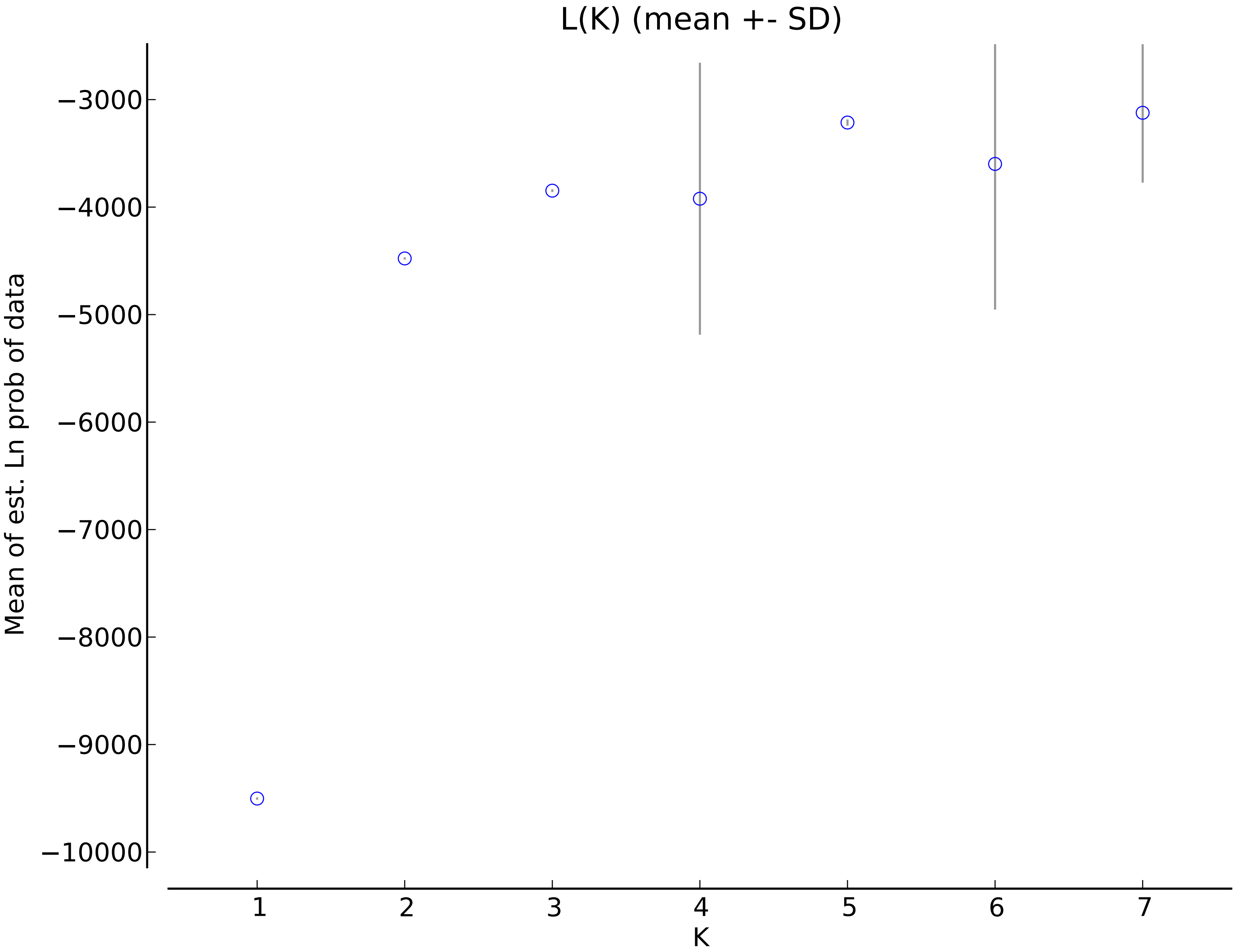

L(K) (mean +- SD)
Mean of est. Ln prob of data
−3000
−4000
−5000
−6000
−7000
−8000
−9000
−10000
1
2
3
4
5
6
7
K
