# Supplementary material for: Do genome size differences within Brachionus asplanchnoidis (Rotifera, Monogononta) cause reproductive barriers among geographic populations?
Source: Hydrobiologia. Author manuscript; Available in PMC 2021 Nov 10. (PMC7611973; doi:10.1007/s10750-016-2872-x)
Supplement: Supplement 2 [file EMS137972-supplement-Supplement_2.zip › 92del_r1/92del_r1.tar.gz!/lnPK.pdf]

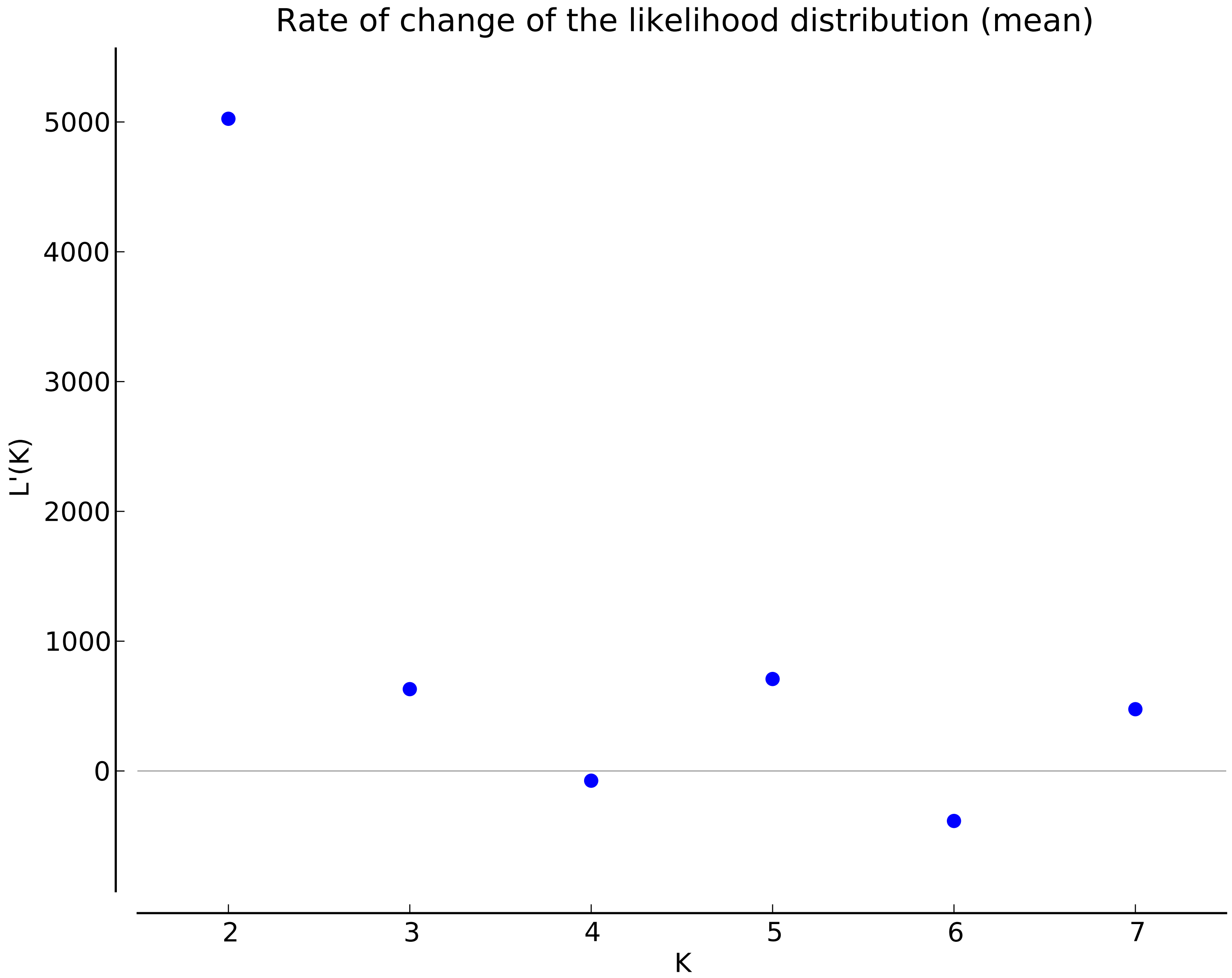
Rate of change of the likelihood distribution (mean)
L'(K)
5000
4000
3000
2000
1000
0
K
2
3
4
5
6
7
